# Supplementary material for: Plant-pollinator interactions along the altitudinal gradient in Berberis lycium royle: An endangered medicinal plant of the Himalayan region
Source: PLoS One. 2025 May 7;20(5):e0310572. doi: 10.1371/journal.pone.0310572 (PMC12058197; doi:10.1371/journal.pone.0310572)
Supplement: Supplementary table S1 — (DOCX) [file pone.0310572.s001.docx]

| **Site 1 (800m) Foraging Behaviour** | | | | | | |
| --- | --- | --- | --- | --- | --- | --- |
| **Species** | **n** | **mean** | **sd** | **min** | **max** | **Range** |
| *Allograpta* sp | 3 | 4.30 | 0.26 | 4.0 | 4.5 | 0.5 |
| *Apis* sp | 3 | 6.17 | 0.21 | 6.0 | 6.4 | 0.4 |
| *Bombus trifasciatus* | 3 | 5.50 | 0.36 | 5.2 | 5.9 | 0.7 |
| *Formica fusca* | 3 | 2.27 | 1.63 | 1.0 | 4.1 | 3.1 |
| *Episyrphus balteatus* | 3 | 3.60 | 0.46 | 3.2 | 4.1 | 0.9 |
| *Eristalis tenax* | 3 | 4.10 | 0.20 | 3.9 | 4.3 | 0.4 |
| *Musca domestica* | 3 | 8.33 | 0.55 | 7.8 | 8.9 | 1.1 |
| *Calliphora vomitoria* | 3 | 8.33 | 1.25 | 6.9 | 9.2 | 2.3 |
| *Chrysotoxum baphyrum* | 3 | 2.60 | 0.46 | 2.2 | 3.1 | 0.9 |
| *Eupeodes luniger* | 3 | 3.87 | 0.35 | 3.5 | 4.2 | 0.7 |
| *Sarchophaga sp* | 3 | 7.73 | 0.57 | 7.1 | 8.2 | 1.1 |
| *Plecia* sp | 3 | 1.47 | 0.38 | 1.2 | 1.9 | 0.7 |
| *Dyscedrus sp* | 3 | 1.73 | 0.47 | 1.2 | 2.1 | 0.9 |
| *Aphidoidea* | 3 | 3.53 | 0.35 | 3.2 | 3.9 | 0.7 |
| *Celastrina argiolus* | 3 | 1.23 | 0.06 | 1.2 | 1.3 | 0.1 |
| *Praezygaena caschmirensis* | 3 | 0.80 | 0.40 | 0.4 | 1.2 | 0.8 |
| *Coccinella undecimpunctata* | 3 | 1.67 | 0.58 | 1.0 | 2.0 | 1.0 |

Supplementary table S1. Showing Foraging Behaviour, Foraging Speed, Insect Visiting Efficiency, Index of Visitation Rate, Density and Relative abundance of various insects individuals species and orders at different altitudes.

| **Site 2 (1150m) Foraging Behaviour** | | | | | | |
| --- | --- | --- | --- | --- | --- | --- |
| **Species** | **n** | **mean** | **sd** | **min** | **max** | **Range** |
| *Apis* sp | 3 | 6.33 | 0.15 | 6.2 | 6.5 | 0.3 |
| *Bombus trifasciatus* | 3 | 5.70 | 1.25 | 4.5 | 7.0 | 2.5 |
| *Formica* *fusca* | 3 | 1.83 | 0.45 | 1.4 | 2.3 | 0.9 |
| *Coelioxys* sp | 3 | 1.77 | 0.25 | 1.5 | 2.0 | 0.5 |
| *Episyrphus balteatus* | 3 | 4.27 | 0.21 | 4.1 | 4.5 | 0.4 |
| *Rhingia* sp | 3 | 3.40 | 0.46 | 2.9 | 3.8 | 0.9 |
| *Eristalis tenax* | 3 | 4.50 | 0.40 | 4.1 | 4.9 | 0.8 |
| *Musca domestica* | 3 | 9.27 | 0.21 | 9.1 | 9.5 | 0.4 |
| *Calliphora vomitoria* | 3 | 9.33 | 0.45 | 8.9 | 9.8 | 0.9 |
| *Calliphora* sp | 3 | 8.33 | 0.50 | 7.8 | 8.8 | 1.0 |
| *Chrysotoxum baphyrum* | 3 | 2.03 | 0.21 | 1.8 | 2.2 | 0.4 |
| *Eupeodes luniger* | 3 | 4.87 | 0.25 | 4.6 | 5.1 | 0.5 |
| *Sarchophaga* sp | 3 | 8.07 | 0.25 | 7.8 | 8.3 | 0.5 |
| *Plecia* sp | 3 | 1.13 | 0.21 | 0.9 | 1.3 | 0.4 |
| *Eristalis cerealis* | 3 | 4.53 | 0.25 | 4.3 | 4.8 | 0.5 |
| *Dyscedrus* sp | 3 | 1.03 | 0.42 | 0.7 | 1.5 | 0.8 |
| *Aphidoidea* | 3 | 2.50 | 0.40 | 2.1 | 2.9 | 0.8 |
| *Praezygaena caschmirensis* | 3 | 0.93 | 0.25 | 0.7 | 1.2 | 0.5 |
| *Celastrina argiolus* | 3 | 1.63 | 0.15 | 1.5 | 1.8 | 0.3 |
| *Altica* sp | 3 | 2.43 | 0.76 | 1.9 | 3.3 | 1.4 |
| *Coccinella undecimpunctata* | 3 | 1.03 | 0.42 | 0.7 | 1.5 | 0.8 |

| **Site 3 (1500m) Foraging Behaviour** | | | | | | |
| --- | --- | --- | --- | --- | --- | --- |
| **Species** | **n** | **mean** | **sd** | **min** | **max** | **Range** |
| *Apis sp* | 3 | 6.33 | 0.45 | 5.9 | 6.8 | 0.9 |
| *Apis cerena* | 3 | 6.60 | 1.25 | 5.3 | 7.8 | 2.5 |
| *Camponotus pennsylvanicus* | 3 | 3.40 | 0.44 | 3.1 | 3.9 | 0.8 |
| *Musca domestica* | 3 | 8.33 | 0.40 | 7.9 | 8.7 | 0.8 |
| *Episyrphus balteatus* | 3 | 6.30 | 1.51 | 4.9 | 7.9 | 3.0 |
| *Eristalis cerealis* | 3 | 4.83 | 0.25 | 4.6 | 5.1 | 0.5 |
| *Eristalis tenax* | 3 | 5.17 | 0.12 | 5.1 | 5.3 | 0.2 |
| *Episyrphys viridaureus* | 3 | 4.43 | 0.50 | 3.9 | 4.9 | 1.0 |
| *Eristalinus taeniops* | 3 | 4.03 | 0.61 | 3.5 | 4.7 | 1.2 |
| *Lucilia* sp | 3 | 6.67 | 1.36 | 5.1 | 7.5 | 2.4 |
| *Rhingia* sp | 3 | 4.67 | 1.50 | 3.1 | 6.1 | 3.0 |
| *Syritta sp* | 3 | 4.30 | 1.93 | 2.9 | 6.5 | 3.6 |
| *Sarcophaga* sp | 3 | 8.37 | 1.31 | 6.9 | 9.4 | 2.5 |
| *Eupeodes luniger* | 3 | 5.33 | 1.85 | 3.5 | 7.2 | 3.7 |
| *Aphidoidea* | 3 | 2.97 | 0.50 | 2.5 | 3.5 | 1.0 |
| *Dodona durga* | 3 | 1.57 | 0.70 | 0.9 | 2.3 | 1.4 |
| *Celastrina argiolus* | 3 | 1.50 | 1.31 | 0.3 | 2.9 | 2.6 |
| *Praezygaena caschmirensis* | 3 | 0.70 | 1.04 | 0.1 | 1.9 | 1.8 |
| *Altica* sp | 3 | 3.30 | 1.71 | 1.9 | 5.2 | 3.3 |

| **Site 4 (1850m) Foraging Behaviour** | | | | | | |
| --- | --- | --- | --- | --- | --- | --- |
| **Species** | **n** | **mean** | **sd** | **min** | **max** | **Range** |
| *Apis* sp | 3 | 6.27 | 1.46 | 4.9 | 7.8 | 2.9 |
| *Apis cerena* | 3 | 6.30 | 0.20 | 6.1 | 6.5 | 0.4 |
| *Bombus* sp | 3 | 6.07 | 1.61 | 4.9 | 7.9 | 3.0 |
| *Bombus haemorrhoidalis* | 3 | 4.70 | 0.75 | 3.9 | 5.4 | 1.5 |
| *Camponotus pennyslyvanicus* | 3 | 3.83 | 0.70 | 3.1 | 4.5 | 1.4 |
| *Eristalis cerealis* | 3 | 5.60 | 1.41 | 4.1 | 6.9 | 2.8 |
| *Eristalis tenax* | 3 | 6.00 | 1.25 | 4.8 | 7.3 | 2.5 |
| *Lucilia* sp | 3 | 7.20 | 0.61 | 6.8 | 7.9 | 1.1 |
| *Musca domestica* | 3 | 9.10 | 1.14 | 7.8 | 9.9 | 2.1 |
| *Episyrphus viridaureus* | 3 | 5.07 | 0.74 | 4.5 | 5.9 | 1.4 |
| *Syritta sp* | 3 | 3.53 | 1.48 | 1.9 | 4.8 | 2.9 |
| *Episyrphus balteatus* | 3 | 6.03 | 1.70 | 4.4 | 7.8 | 3.4 |
| *Celastrina argiolus* | 3 | 1.67 | 1.31 | 0.3 | 2.9 | 2.6 |
| *Heliophorus moorei* | 3 | 0.97 | 0.83 | 0.3 | 1.9 | 1.6 |
| *Celastrina* sp | 3 | 1.97 | 1.68 | 0.9 | 3.9 | 3.0 |
| *Heliophorus sena* | 3 | 2.50 | 1.54 | 0.8 | 3.8 | 3.0 |
| *Heliophorus* sp | 3 | 1.33 | 1.40 | 0.2 | 2.9 | 2.7 |
| *Cyrestis thyodamas* | 3 | 2.17 | 1.52 | 0.8 | 3.8 | 3.0 |
| *Dodona durga* | 3 | 1.67 | 1.27 | 0.3 | 2.8 | 2.5 |
| *Aglais caschmirensis* | 3 | 3.23 | 0.42 | 2.9 | 3.7 | 0.8 |
| *Coccinella undecimpunctata* | 3 | 1.83 | 1.30 | 0.5 | 3.1 | 2.6 |
| *Coccinella septempunctata* | 3 | 1.77 | 1.33 | 0.3 | 2.9 | 2.6 |

| **Site 5 (2200m) Foraging Behaviour** | | | | | | |
| --- | --- | --- | --- | --- | --- | --- |
| **Species** | **n** | **mean** | **sd** | **min** | **max** | **Range** |
| *Apis* sp | 3 | 5.50 | 0.87 | 4.5 | 6.1 | 1.6 |
| *Bombus* sp | 3 | 5.33 | 0.55 | 4.8 | 5.9 | 1.1 |
| *Camponotus pennyslyvanicus* | 3 | 3.77 | 1.33 | 2.9 | 5.3 | 2.4 |
| *Eristalis cerealis* | 3 | 6.07 | 1.46 | 4.9 | 7.7 | 2.8 |
| *Eristalis tenax* | 3 | 5.67 | 0.91 | 4.7 | 6.5 | 1.8 |
| *Musca domestica* | 3 | 8.70 | 1.31 | 7.3 | 9.9 | 2.6 |
| *Episyrphus viridaureus* | 3 | 4.77 | 0.99 | 4.1 | 5.9 | 1.8 |
| *Episyrphus balteatus* | 3 | 5.93 | 0.91 | 5.1 | 6.9 | 1.8 |
| *Lucilia* sp | 3 | 7.47 | 1.50 | 5.9 | 8.9 | 3.0 |
| *Syritta* sp | 3 | 3.93 | 1.82 | 1.9 | 5.4 | 3.5 |
| *Celastrina argiolus* | 3 | 2.60 | 1.65 | 0.9 | 4.2 | 3.3 |
| *Heliophorus moorei* | 3 | 0.83 | 0.50 | 0.3 | 1.3 | 1.0 |
| *Heliophorus sena* | 3 | 1.57 | 0.76 | 0.7 | 2.1 | 1.4 |
| *Cyrestis thyodamas* | 3 | 2.60 | 1.77 | 0.7 | 4.2 | 3.5 |
| *Dodona durga* | 3 | 2.03 | 1.33 | 0.9 | 3.5 | 2.6 |
| *Aglais caschmirensis* | 3 | 0.70 | 0.20 | 0.5 | 0.9 | 0.4 |
| *Pieris* sp | 3 | 1.40 | 0.26 | 1.2 | 1.7 | 0.5 |
| *Coccinella septempunctata* | 3 | 2.43 | 1.50 | 0.9 | 3.9 | 3.0 |
| *Coccinella undecimpunctata* | 3 | 2.23 | 1.76 | 0.4 | 3.9 | 3.5 |

| **Site 1 (800m) Foraging Speed** | | | | | | |
| --- | --- | --- | --- | --- | --- | --- |
| **Species** | **n** | **mean** | **Sd** | **min** | **max** | **Range** |
| *Allograpta* sp | 3 | 31.63 | 2.12 | 29.9 | 34.0 | 4.1 |
| *Apis* sp | 3 | 40.23 | 4.52 | 37.0 | 45.4 | 8.4 |
| *Bombus trifasciatus* | 3 | 48.93 | 6.82 | 41.2 | 54.1 | 12.9 |
| *Formica fusca* | 3 | 39.70 | 0.53 | 8.9 | 9.9 | 1.0 |
| *Episyrphus balteatus* | 3 | 3.60 | 0.72 | 39.1 | 40.5 | 1.4 |
| *Eristalis tenax* | 3 | 46.60 | 5.60 | 40.5 | 51.5 | 11.0 |
| *Musca domestica* | 3 | 41.27 | 1.11 | 40.1 | 42.3 | 2.2 |
| *Calliphora vomitoria* | 3 | 51.50 | 1.48 | 50.5 | 53.2 | 2.7 |
| *Chrysotoxum baphyrum* | 3 | 12.40 | 2.39 | 10.3 | 15.0 | 4.7 |
| *Eupeodes luniger* | 3 | 45.93 | 5.83 | 39.2 | 49.5 | 10.3 |
| *Sarchophaga sp* | 3 | 45.67 | 2.14 | 44.1 | 48.1 | 4.0 |
| *Plecia* sp | 3 | 12.70 | 1.40 | 11.3 | 14.1 | 2.8 |
| *Dyscedrus sp* | 3 | 11.70 | 1.83 | 9.7 | 13.3 | 3.6 |
| *Aphidoidea* | 3 | 20.63 | 2.25 | 18.7 | 23.1 | 4.4 |
| *Celastrina argiolus* | 3 | 39.00 | 3.00 | 36.1 | 42.1 | 6.0 |
| *Praezygaena caschmirensis* | 3 | 25.13 | 1.81 | 23.8 | 27.2 | 3.4 |
| *Coccinella undecimpunctata* | 3 | 7.57 | 2.07 | 5.7 | 9.8 | 4.1 |

| **Site 2 (1150m) Foraging Speed** | | | | | | |
| --- | --- | --- | --- | --- | --- | --- |
| **Species** | **n** | **mean** | **sd** | **min** | **max** | **Range** |
| *Apis* sp | 3 | 48.07 | 1.80 | 46.7 | 50.1 | 3.4 |
| *Bombus trifasciatus* | 3 | 46.60 | 1.55 | 45.0 | 48.1 | 3.1 |
| *Formica* *fusca* | 3 | 15.43 | 1.89 | 13.3 | 16.9 | 3.6 |
| *Coelioxys* sp | 3 | 15.33 | 1.96 | 13.5 | 17.4 | 3.9 |
| *Episyrphus balteatus* | 3 | 42.43 | 0.95 | 41.7 | 43.5 | 1.8 |
| *Rhingia* sp | 3 | 15.40 | 1.01 | 14.5 | 16.5 | 2.0 |
| *Eristalis tenax* | 3 | 45.57 | 10.41 | 37.8 | 57.4 | 19.6 |
| *Musca domestica* | 3 | 46.57 | 4.02 | 42.1 | 49.9 | 7.8 |
| *Calliphora vomitoria* | 3 | 53.33 | 2.68 | 50.9 | 56.2 | 5.3 |
| *Calliphora* sp | 3 | 50.07 | 1.45 | 48.6 | 51.5 | 2.9 |
| *Chrysotoxum baphyrum* | 3 | 17.07 | 1.96 | 15.0 | 18.9 | 3.9 |
| *Eupeodes luniger* | 3 | 29.87 | 1.93 | 27.7 | 31.4 | 3.7 |
| *Sarchophaga* sp | 3 | 42.43 | 2.84 | 40.1 | 45.6 | 5.5 |
| *Plecia* sp | 3 | 19.20 | 1.77 | 17.3 | 20.8 | 3.5 |
| *Eristalis cerealis* | 3 | 44.77 | 2.35 | 42.4 | 47.1 | 4.7 |
| *Dyscedrus* sp | 3 | 15.23 | 4.48 | 10.5 | 19.4 | 8.9 |
| *Aphidoidea* | 3 | 23.03 | 1.77 | 21.0 | 24.2 | 3.2 |
| *Praezygaena caschmirensis* | 3 | 18.20 | 1.15 | 16.9 | 19.1 | 2.2 |
| *Celastrina argiolus* | 3 | 15.43 | 1.27 | 14.3 | 16.8 | 2.5 |
| *Altica* sp | 3 | 13.97 | 2.44 | 11.3 | 16.1 | 4.8 |
| *Coccinella undecimpunctata* | 3 | 7.03 | 1.07 | 6.1 | 8.2 | 2.1 |

| **Site 3 (1500m) Foraging Speed** | | | | | | |
| --- | --- | --- | --- | --- | --- | --- |
| **Species** | **n** | **mean** | **sd** | **min** | **max** | **Range** |
| *Apis sp* | 3 | 40.00 | 1.80 | 38.0 | 41.5 | 3.5 |
| *Apis cerena* | 3 | 34.37 | 2.10 | 32.2 | 36.4 | 4.2 |
| *Camponotus pennsylvanicus* | 3 | 7.80 | 1.05 | 6.8 | 8.9 | 2.1 |
| *Musca domestica* | 3 | 49.33 | 1.43 | 48.1 | 50.9 | 2.8 |
| *Episyrphus balteatus* | 3 | 45.20 | 1.87 | 43.2 | 46.9 | 3.7 |
| *Eristalis cerealis* | 3 | 41.67 | 1.96 | 39.8 | 43.7 | 3.9 |
| *Eristalis tenax* | 3 | 43.50 | 0.92 | 42.5 | 44.3 | 1.8 |
| *Episyrphys viridaureus* | 3 | 39.80 | 1.70 | 38.1 | 41.5 | 3.4 |
| *Eristalinus taeniops* | 3 | 40.90 | 1.51 | 39.5 | 42.5 | 3.0 |
| *Lucilia* sp | 3 | 24.63 | 1.15 | 23.5 | 25.8 | 2.3 |
| *Rhingia* sp | 3 | 10.23 | 0.74 | 9.4 | 10.8 | 1.4 |
| *Syritta sp* | 3 | 42.47 | 1.63 | 40.7 | 43.9 | 3.2 |
| *Sarcophaga* sp | 3 | 42.80 | 2.30 | 40.5 | 45.1 | 4.6 |
| *Eupeodes luniger* | 3 | 31.00 | 1.32 | 30.0 | 32.2 | 2.5 |
| *Aphidoidea* | 3 | 17.87 | 1.81 | 16.2 | 19.8 | 3.6 |
| *Dodona durga* | 3 | 21.57 | 1.68 | 20.1 | 23.4 | 3.3 |
| *Celastrina argiolus* | 3 | 16.40 | 0.96 | 15.3 | 17.1 | 1.8 |
| *Praezygaena caschmirensis* | 3 | 26.10 | 2.86 | 23.4 | 29.1 | 5.7 |
| *Altica* sp | 3 | 15.70 | 1.85 | 13.9 | 17.6 | 3.7 |

| **Site 4 (1850m) Foraging Speed** | | | | | | |
| --- | --- | --- | --- | --- | --- | --- |
| **Species** | **n** | **mean** | **sd** | **min** | **max** | **Range** |
| *Apis* sp | 3 | 37.20 | 0.75 | 36.5 | 38.0 | 1.5 |
| *Apis cerena* | 3 | 36.60 | 1.35 | 35.2 | 37.9 | 2.7 |
| *Bombus* sp | 3 | 39.53 | 0.60 | 38.0 | 40.1 | 1.2 |
| *Bombus haemorrhoidalis* | 3 | 41.93 | 1.27 | 40.5 | 42.9 | 2.4 |
| *Camponotus pennyslyvanicus* | 3 | 11.70 | 1.25 | 10.4 | 12.9 | 2.5 |
| *Eristalis cerealis* | 3 | 38.40 | 1.06 | 37.2 | 39.2 | 2.0 |
| *Eristalis tenax* | 3 | 41.83 | 1.50 | 40.4 | 43.4 | 3.0 |
| *Lucilia* sp | 3 | 22.50 | 0.89 | 21.8 | 23.5 | 1.7 |
| *Musca domestica* | 3 | 44.93 | 0.75 | 44.2 | 45.7 | 1.5 |
| *Episyrphus viridaureus* | 3 | 37.87 | 0.46 | 37.6 | 38.4 | 0.8 |
| *Syritta sp* | 3 | 6.80 | 0.96 | 6.1 | 7.9 | 1.8 |
| *Episyrphus balteatus* | 3 | 47.87 | 1.16 | 46.8 | 49.1 | 2.3 |
| *Celastrina argiolus* | 3 | 10.40 | 0.36 | 10.1 | 10.8 | 0.7 |
| *Heliophorus moorei* | 3 | 20.77 | 0.49 | 20.2 | 21.1 | 0.9 |
| *Celastrina* sp | 3 | 17.67 | 0.86 | 16.9 | 18.6 | 1.7 |
| *Heliophorus sena* | 3 | 14.37 | 0.47 | 14.0 | 14.9 | 0.9 |
| *Heliophorus* sp | 3 | 10.67 | 0.60 | 10.1 | 11.3 | 1.2 |
| *Cyrestis thyodamas* | 3 | 14.50 | 0.44 | 14.0 | 14.8 | 0.8 |
| *Dodona durga* | 3 | 24.50 | 0.46 | 24.0 | 24.9 | 0.9 |
| *Aglais caschmirensis* | 3 | 9.40 | 0.62 | 8.9 | 10.1 | 1.2 |
| *Coccinella undecimpunctata* | 3 | 8.23 | 0.31 | 7.9 | 8.5 | 0.6 |
| *Coccinella septempunctata* | 3 | 8.07 | 0.76 | 7.2 | 8.6 | 1.4 |

| **Site 5 (2200m) Foraging Speed** | | | | | | |
| --- | --- | --- | --- | --- | --- | --- |
| **Species** | **n** | **mean** | **sd** | **min** | **max** | **Range** |
| *Apis* sp | 3 | 40.83 | 2.39 | 38.9 | 43.5 | 4.6 |
| *Bombus* sp | 3 | 40.57 | 0.51 | 40.0 | 41.0 | 1.0 |
| *Camponotus pennyslyvanicus* | 3 | 12.60 | 0.62 | 11.9 | 13.1 | 1.2 |
| *Eristalis cerealis* | 3 | 40.47 | 1.60 | 38.9 | 42.1 | 3.2 |
| *Eristalis tenax* | 3 | 38.40 | 1.11 | 37.2 | 39.4 | 2.2 |
| *Musca domestica* | 3 | 45.73 | 1.60 | 44.2 | 47.4 | 3.2 |
| *Episyrphus viridaureus* | 3 | 45.20 | 0.87 | 44.2 | 45.8 | 1.6 |
| *Episyrphus balteatus* | 3 | 41.17 | 0.86 | 40.4 | 42.1 | 1.7 |
| *Lucilia* sp | 3 | 41.50 | 1.59 | 39.7 | 42.7 | 3.0 |
| *Syritta* sp | 3 | 6.93 | 0.40 | 6.5 | 7.3 | 0.8 |
| *Celastrina argiolus* | 3 | 13.30 | 0.56 | 12.7 | 13.8 | 1.1 |
| *Heliophorus moorei* | 3 | 10.90 | 0.56 | 10.4 | 11.5 | 1.1 |
| *Heliophorus sena* | 3 | 14.23 | 0.45 | 13.8 | 14.7 | 0.9 |
| *Cyrestis thyodamas* | 3 | 15.47 | 0.91 | 14.8 | 16.5 | 1.7 |
| *Dodona durga* | 3 | 19.13 | 0.40 | 18.7 | 19.5 | 0.8 |
| *Aglais caschmirensis* | 3 | 6.90 | 1.30 | 5.6 | 8.2 | 2.6 |
| *Pieris* sp | 3 | 8.70 | 1.21 | 7.4 | 9.8 | 2. |
| *Coccinella septempunctata* | 3 | 6.80 | 1.39 | 6.0 | 8.4 | 2.4 |
| *Coccinella undecimpunctata* | 3 | 5.93 | 0.81 | 5.2 | 6.8 | 1.6 |

| **Site 1 (800m) Insect Visiting Efficiency** | | | | | | |
| --- | --- | --- | --- | --- | --- | --- |
| **Species** | **n** | **mean** | **sd** | **min** | **max** | **Range** |
| *Allograpta* sp | 3 | 0.12 | 0.01 | 0.11 | 0.12 | 0.01 |
| *Apis* sp | 3 | 0.18 | 0.02 | 0.17 | 0.21 | 0.04 |
| *Bombus trifasciatus* | 3 | 0.25 | 0.04 | 0.21 | 0.28 | 0.07 |
| *Formica fusca* | 3 | 0.03 | 0.00 | 0.03 | 0.03 | 0.00 |
| *Episyrphus balteatus* | 3 | 0.19 | 0.00 | 0.19 | 0.19 | 0.01 |
| *Eristalis tenax* | 3 | 0.15 | 0.01 | 0.14 | 0.16 | 0.02 |
| *Musca domestica* | 3 | 0.22 | 0.01 | 0.21 | 0.22 | 0.01 |
| *Calliphora vomitoria* | 3 | 0.34 | 0.01 | 0.33 | 0.35 | 0.02 |
| *Chrysotoxum baphyrum* | 3 | 0.04 | 0.01 | 0.03 | 0.04 | 0.01 |
| *Eupeodes luniger* | 3 | 0.12 | 0.02 | 0.10 | 0.13 | 0.03 |
| *Sarchophaga sp* | 3 | 0.22 | 0.01 | 0.21 | 0.23 | 0.02 |
| *Plecia* sp | 3 | 0.03 | 0.00 | 0.03 | 0.04 | 0.01 |
| *Dyscedrus sp* | 3 | 0.03 | 0.00 | 0.03 | 0.04 | 0.01 |
| *Aphidoidea* | 3 | 0.06 | 0.01 | 0.06 | 0.07 | 0.01 |
| *Celastrina argiolus* | 3 | 0.09 | 0.01 | 0.08 | 0.10 | 0.01 |
| *Praezygaena caschmirensis* | 3 | 0.06 | 0.00 | 0.06 | 0.07 | 0.01 |
| *Coccinella undecimpunctata* | 3 | 0.02 | 0.01 | 0.01 | 0.02 | 0.01 |

| **Site 2 (1150m) Insect Visiting Efficiency** | | | | | | |
| --- | --- | --- | --- | --- | --- | --- |
| **Species** | **n** | **mean** | **sd** | **min** | **max** | **Range** |
| *Apis* sp | 3 | 0.21 | 0.01 | 0.21 | 0.22 | 0.01 |
| *Bombus trifasciatus* | 3 | 0.24 | 0.01 | 0.23 | 0.02 | 0.02 |
| *Formica* *fusca* | 3 | 0.04 | 0.00 | 0.03 | 0.01 | 0.01 |
| *Coelioxys* sp | 3 | 0.06 | 0.01 | 0.05 | 0.02 | 0.02 |
| *Episyrphus balteatus* | 3 | 0.20 | 0.00 | 0.20 | 0.01 | 0.01 |
| *Rhingia* sp | 3 | 0.04 | 0.00 | 0.04 | 0.04 | 0.00 |
| *Eristalis tenax* | 3 | 0.17 | 0.04 | 0.14 | 0.22 | 0.08 |
| *Musca domestica* | 3 | 0.23 | 0.02 | 0.21 | 0.24 | 0.03 |
| *Calliphora vomitoria* | 3 | 0.25 | 0.00 | 0.25 | 0.26 | 0.01 |
| *Calliphora* sp | 3 | 0.28 | 0.00 | 0.28 | 0.29 | 0.01 |
| *Chrysotoxum baphyrum* | 3 | 0.05 | 0.01 | 0.04 | 0.05 | 0.01 |
| *Eupeodes luniger* | 3 | 0.12 | 0.01 | 0.11 | 0.12 | 0.01 |
| *Sarchophaga* sp | 3 | 0.13 | 0.01 | 0.13 | 0.14 | 0.01 |
| *Plecia* sp | 3 | 0.04 | 0.00 | 0.04 | 0.05 | 0.01 |
| *Eristalis cerealis* | 3 | 0.20 | 0.01 | 0.19 | 0.21 | 0.01 |
| *Dyscedrus* sp | 3 | 0.04 | 0.01 | 0.03 | 0.05 | 0.02 |
| *Aphidoidea* | 3 | 0.06 | 0.00 | 0.06 | 0.07 | 0.01 |
| *Praezygaena caschmirensis* | 3 | 0.06 | 0.00 | 0.06 | 0.06 | 0.00 |
| *Celastrina argiolus* | 3 | 0.04 | 0.00 | 0.04 | 0.05 | 0.01 |
| *Altica* sp | 3 | 0.03 | 0.01 | 0.03 | 0.04 | 0.01 |
| *Coccinella undecimpunctata* | 3 | 0.02 | 0.00 | 0.01 | 0.02 | 0.00 |

| **Site 3 (1500m) Insect Visiting Efficiency** | | | | | | |
| --- | --- | --- | --- | --- | --- | --- |
| **Species** | **n** | **mean** | **sd** | **min** | **max** | **Range** |
| *Apis sp* | 3 | 0.22 | 0.01 | 0.20 | 0.23 | 0.03 |
| *Apis cerena* | 3 | 0.20 | 0.01 | 0.19 | 0.21 | 0.02 |
| *Camponotus pennsylvanicus* | 3 | 0.03 | 0.00 | 0.03 | 0.03 | 0.01 |
| *Musca domestica* | 3 | 0.29 | 0.00 | 0.28 | 0.29 | 0.01 |
| *Episyrphus balteatus* | 3 | 0.20 | 0.01 | 0.20 | 0.21 | 0.01 |
| *Eristalis cerealis* | 3 | 0.18 | 0.01 | 0.17 | 0.19 | 0.02 |
| *Eristalis tenax* | 3 | 0.23 | 0.00 | 0.22 | 0.23 | 0.00 |
| *Episyrphys viridaureus* | 3 | 0.20 | 0.01 | 0.19 | 0.20 | 0.01 |
| *Eristalinus taeniops* | 3 | 0.22 | 0.00 | 0.22 | 0.22 | 0.01 |
| *Lucilia* sp | 3 | 0.14 | 0.00 | 0.13 | 0.14 | 0.01 |
| *Rhingia* sp | 3 | 0.04 | 0.00 | 0.03 | 0.04 | 0.00 |
| *Syritta sp* | 3 | 0.17 | 0.01 | 0.17 | 0.18 | 0.01 |
| *Sarcophaga* sp | 3 | 0.23 | 0.01 | 0.22 | 0.25 | 0.02 |
| *Eupeodes luniger* | 3 | 0.13 | 0.01 | 0.13 | 0.14 | 0.01 |
| *Aphidoidea* | 3 | 0.08 | 0.01 | 0.07 | 0.09 | 0.01 |
| *Dodona durga* | 3 | 0.10 | 0.01 | 0.09 | 0.10 | 0.01 |
| *Celastrina argiolus* | 3 | 0.05 | 0.00 | 0.05 | 0.06 | 0.01 |
| *Praezygaena caschmirensis* | 3 | 0.08 | 0.01 | 0.07 | 0.09 | 0.02 |
| *Altica* sp | 3 | 0.04 | 0.00 | 0.03 | 0.04 | 0.01 |

| **Site 4 (1850m) Insect Visiting Efficiency** | | | | | | |
| --- | --- | --- | --- | --- | --- | --- |
| **Species** | **n** | **mean** | **sd** | **min** | **max** | **Range** |
| *Apis* sp | 3 | 0.20 | 0.00 | 0.20 | 0.20 | 0.01 |
| *Apis cerena* | 3 | 0.21 | 0.01 | 0.20 | 0.21 | 0.01 |
| *Bombus* sp | 3 | 0.24 | 0.03 | 0.21 | 0.26 | 0.05 |
| *Bombus haemorrhoidalis* | 3 | 0.22 | 0.01 | 0.22 | 0.23 | 0.01 |
| *Camponotus pennyslyvanicus* | 3 | 0.04 | 0.00 | 0.03 | 0.04 | 0.01 |
| *Eristalis cerealis* | 3 | 0.24 | 0.00 | 0.23 | 0.24 | 0.01 |
| *Eristalis tenax* | 3 | 0.26 | 0.01 | 0.25 | 0.27 | 0.02 |
| *Lucilia* sp | 3 | 0.13 | 0.00 | 0.13 | 0.13 | 0.01 |
| *Musca domestica* | 3 | 0.24 | 0.00 | 0.24 | 0.24 | 0.01 |
| *Episyrphus viridaureus* | 3 | 0.22 | 0.00 | 0.22 | 0.22 | 0.00 |
| *Syritta sp* | 3 | 0.02 | 0.00 | 0.02 | 0.02 | 0.00 |
| *Episyrphus balteatus* | 3 | 0.21 | 0.00 | 0.20 | 0.21 | 0.01 |
| *Celastrina argiolus* | 3 | 0.04 | 0.00 | 0.04 | 0.05 | 0.00 |
| *Heliophorus moorei* | 3 | 0.06 | 0.00 | 0.06 | 0.06 | 0.00 |
| *Celastrina* sp | 3 | 0.04 | 0.00 | 0.04 | 0.04 | 0.00 |
| *Heliophorus sena* | 3 | 0.04 | 0.00 | 0.04 | 0.04 | 0.00 |
| *Heliophorus* sp | 3 | 0.04 | 0.00 | 0.04 | 0.04 | 0.00 |
| *Cyrestis thyodamas* | 3 | 0.03 | 0.00 | 0.03 | 0.03 | 0.00 |
| *Dodona durga* | 3 | 0.09 | 0.00 | 0.09 | 0.09 | 0.00 |
| *Aglais caschmirensis* | 3 | 0.02 | 0.00 | 0.02 | 0.02 | 0.00 |
| *Coccinella undecimpunctata* | 3 | 0.02 | 0.00 | 0.02 | 0.02 | 0.00 |
| *Coccinella septempunctata* | 3 | 0.02 | 0.00 | 0.02 | 0.02 | 0.00 |

| **Site 5 (2200m) Insect Visiting Efficiency** | | | | | | |
| --- | --- | --- | --- | --- | --- | --- |
| **Species** | **n** | **mean** | **sd** | **min** | **max** | **Range** |
| *Apis* sp | 3 | 0.22 | 0.01 | 0.21 | 0.23 | 0.02 |
| *Bombus* sp | 3 | 0.21 | 0.00 | 0.20 | 0.21 | 0.00 |
| *Camponotus pennyslyvanicus* | 3 | 0.04 | 0.00 | 0.04 | 0.04 | 0.00 |
| *Eristalis cerealis* | 3 | 0.24 | 0.01 | 0.24 | 0.25 | 0.01 |
| *Eristalis tenax* | 3 | 0.22 | 0.01 | 0.22 | 0.23 | 0.01 |
| *Musca domestica* | 3 | 0.30 | 0.01 | 0.29 | 0.30 | 0.02 |
| *Episyrphus viridaureus* | 3 | 0.21 | 0.00 | 0.20 | 0.21 | 0.00 |
| *Episyrphus balteatus* | 3 | 0.23 | 0.00 | 0.23 | 0.24 | 0.01 |
| *Lucilia* sp | 3 | 0.14 | 0.00 | 0.13 | 0.14 | 0.01 |
| *Syritta* sp | 3 | 0.02 | 0.00 | 0.02 | 0.02 | 0.00 |
| *Celastrina argiolus* | 3 | 0.04 | 0.00 | 0.04 | 0.04 | 0.00 |
| *Heliophorus moorei* | 3 | 0.03 | 0.00 | 0.03 | 0.03 | 0.00 |
| *Heliophorus sena* | 3 | 0.04 | 0.00 | 0.04 | 0.04 | 0.00 |
| *Cyrestis thyodamas* | 3 | 0.05 | 0.00 | 0.05 | 0.05 | 0.01 |
| *Dodona durga* | 3 | 0.09 | 0.00 | 0.09 | 0.09 | 0.00 |
| *Aglais caschmirensis* | 3 | 0.03 | 0.00 | 0.02 | 0.03 | 0.01 |
| *Pieris* sp | 3 | 0.02 | 0.00 | 0.02 | 0.03 | 0.01 |
| *Coccinella septempunctata* | 3 | 0.02 | 0.00 | 0.02 | 0.02 | 0.01 |
| *Coccinella undecimpunctata* | 3 | 0.02 | 0.00 | 0.01 | 0.02 | 0.00 |

| **Site 1 (800m) Index of Visitation Rate** | | | | | | |
| --- | --- | --- | --- | --- | --- | --- |
| **Species** | **n** | **mean** | **sd** | **min** | **max** | **Range** |
| *Allograpta* sp | 3 | 3.62 | 0.24 | 3.42 | 3.89 | 0.47 |
| *Apis* sp | 3 | 38.39 | 4.31 | 35.31 | 43.32 | 8.02 |
| *Bombus trifasciatus* | 3 | 6.91 | 3.98 | 2.54 | 10.32 | 7.78 |
| *Formica fusca* | 3 | 0.35 | 0.02 | 0.34 | 0.38 | 0.04 |
| *Episyrphus balteatus* | 3 | 53.03 | 0.96 | 52.23 | 54.10 | 1.87 |
| *Eristalis tenax* | 3 | 51.58 | 6.20 | 44.83 | 57.00 | 12.18 |
| *Musca domestica* | 3 | 39.38 | 1.06 | 38.26 | 40.36 | 2.10 |
| *Calliphora vomitoria* | 3 | 68.80 | 1.98 | 67.46 | 71.07 | 3.61 |
| *Chrysotoxum baphyrum* | 3 | 11.36 | 2.19 | 9.44 | 13.74 | 4.31 |
| *Eupeodes luniger* | 3 | 52.60 | 6.68 | 44.89 | 56.68 | 11.79 |
| *Sarchophaga sp* | 3 | 26.15 | 1.22 | 25.25 | 27.54 | 2.29 |
| *Plecia* sp | 3 | 2.42 | 0.27 | 2.16 | 2.69 | 0.53 |
| *Dyscedrus sp* | 3 | 4.02 | 0.63 | 3.33 | 4.57 | 1.24 |
| *Aphidoidea* | 3 | 6.30 | 0.69 | 5.71 | 7.05 | 1.34 |
| *Celastrina argiolus* | 3 | 5.95 | 0.46 | 5.51 | 6.43 | 0.92 |
| *Praezygaena caschmirensis* | 3 | 4.80 | 0.35 | 4.54 | 5.19 | 0.65 |
| *Coccinella undecimpunctata* | 3 | 0.87 | 0.24 | 0.65 | 1.12 | 0.47 |

| **Site 2 (1150m) Index of Visitation Rate** | | | | | | |
| --- | --- | --- | --- | --- | --- | --- |
| **Species** | **n** | **mean** | **Sd** | **min** | **max** | **Range** |
| *Apis* sp | 3 | 45.37 | 1.69 | 44.08 | 47.29 | 3.21 |
| *Bombus trifasciatus* | 3 | 8.30 | 0.29 | 7.96 | 8.51 | 0.55 |
| *Formica* *fusca* | 3 | 1.37 | 0.17 | 1.18 | 1.50 | 0.32 |
| *Coelioxys* sp | 3 | 1.81 | 0.23 | 1.59 | 2.05 | 0.46 |
| *Episyrphus balteatus* | 3 | 45.06 | 1.00 | 44.28 | 46.19 | 1.91 |
| *Rhingia* sp | 3 | 10.90 | 0.72 | 10.27 | 11.68 | 1.42 |
| *Eristalis tenax* | 3 | 16.13 | 3.69 | 13.38 | 20.32 | 6.94 |
| *Musca domestica* | 3 | 32.97 | 2.85 | 29.81 | 35.33 | 5.52 |
| *Calliphora vomitoria* | 3 | 66.08 | 3.32 | 63.06 | 69.63 | 6.57 |
| *Calliphora* sp | 3 | 45.78 | 1.33 | 44.44 | 47.09 | 2.65 |
| *Chrysotoxum baphyrum* | 3 | 3.02 | 0.35 | 2.65 | 3.35 | 0.69 |
| *Eupeodes luniger* | 3 | 17.62 | 1.14 | 16.34 | 18.53 | 2.18 |
| *Sarchophaga* sp | 3 | 16.27 | 1.09 | 15.38 | 17.49 | 2.11 |
| *Plecia* sp | 3 | 7.36 | 0.68 | 6.63 | 7.98 | 1.34 |
| *Eristalis cerealis* | 3 | 52.82 | 2.77 | 50.03 | 55.58 | 5.55 |
| *Dyscedrus* sp | 3 | 1.35 | 0.40 | 0.93 | 1.72 | 0.79 |
| *Aphidoidea* | 3 | 6.12 | 0.47 | 5.58 | 6.42 | 0.85 |
| *Praezygaena caschmirensis* | 3 | 4.10 | 1.21 | 2.74 | 5.07 | 2.33 |
| *Celastrina argiolus* | 3 | 3.64 | 0.30 | 3.37 | 3.96 | 0.59 |
| *Altica* sp | 3 | 1.65 | 0.29 | 1.33 | 1.90 | 0.57 |
| *Coccinella undecimpunctata* | 3 | 0.83 | 0.13 | 0.72 | 0.97 | 0.25 |

| **Site 3 (1500m) Index of Visitation Rate** | | | | | | |
| --- | --- | --- | --- | --- | --- | --- |
| **Species** | **n** | **mean** | **sd** | **min** | **max** | **Range** |
| *Apis sp* | 3 | 25.38 | 3.04 | 22.69 | 28.67 | 5.99 |
| *Apis cerena* | 3 | 17.95 | 1.10 | 16.82 | 19.01 | 2.19 |
| *Camponotus pennsylvanicus* | 3 | 4.27 | 0.58 | 3.72 | 4.87 | 1.15 |
| *Musca domestica* | 3 | 49.09 | 1.42 | 47.86 | 50.65 | 2.79 |
| *Episyrphus balteatus* | 3 | 37.10 | 1.53 | 35.46 | 38.50 | 3.04 |
| *Eristalis cerealis* | 3 | 18.66 | 0.88 | 17.82 | 19.57 | 1.75 |
| *Eristalis tenax* | 3 | 43.28 | 0.91 | 42.29 | 44.08 | 1.79 |
| *Episyrphys viridaureus* | 3 | 44.55 | 1.90 | 42.65 | 46.46 | 3.81 |
| *Eristalinus taeniops* | 3 | 28.49 | 1.05 | 27.51 | 29.60 | 2.09 |
| *Lucilia* sp | 3 | 20.22 | 0.94 | 19.29 | 21.18 | 1.89 |
| *Rhingia* sp | 3 | 2.29 | 0.17 | 2.10 | 2.42 | 0.31 |
| *Syritta sp* | 3 | 25.35 | 0.97 | 24.30 | 26.21 | 1.91 |
| *Sarcophaga* sp | 3 | 29.81 | 1.60 | 28.21 | 31.41 | 3.20 |
| *Eupeodes luniger* | 3 | 3.86 | 0.16 | 3.73 | 4.04 | 0.31 |
| *Aphidoidea* | 3 | 4.44 | 0.45 | 4.03 | 4.93 | 0.90 |
| *Dodona durga* | 3 | 5.36 | 0.42 | 5.00 | 5.82 | 0.82 |
| *Celastrina argiolus* | 3 | 1.22 | 0.07 | 1.14 | 1.28 | 0.13 |
| *Praezygaena caschmirensis* | 3 | 1.30 | 0.14 | 1.16 | 1.45 | 0.28 |
| *Altica* sp | 3 | 2.73 | 0.32 | 2.42 | 3.06 | 0.64 |

| **Site 4 (1850m) Index of Visitation Rate** | | | | | | |
| --- | --- | --- | --- | --- | --- | --- |
| **Species** | **n** | **mean** | **sd** | **min** | **max** | **Range** |
| *Apis* sp | 3 | 19.10 | 0.39 | 18.74 | 19.51 | 0.77 |
| *Apis cerena* | 3 | 15.78 | 0.58 | 15.18 | 16.34 | 1.16 |
| *Bombus* sp | 3 | 8.93 | 0.14 | 8.79 | 9.06 | 0.27 |
| *Bombus haemorrhoidalis* | 3 | 16.36 | 0.49 | 15.80 | 16.74 | 0.94 |
| *Camponotus pennyslyvanicus* | 3 | 6.01 | 0.64 | 5.34 | 6.62 | 1.28 |
| *Eristalis cerealis* | 3 | 14.19 | 0.39 | 13.75 | 14.49 | 0.74 |
| *Eristalis tenax* | 3 | 21.48 | 0.77 | 20.74 | 22.28 | 1.54 |
| *Lucilia* sp | 3 | 16.63 | 0.66 | 16.11 | 17.37 | 1.26 |
| *Musca domestica* | 3 | 36.91 | 0.62 | 36.30 | 37.54 | 1.23 |
| *Episyrphus viridaureus* | 3 | 20.99 | 0.26 | 20.85 | 21.29 | 0.44 |
| *Syritta sp* | 3 | 4.33 | 0.61 | 3.88 | 5.03 | 1.15 |
| *Episyrphus balteatus* | 3 | 28.50 | 0.69 | 27.87 | 29.24 | 1.37 |
| *Celastrina argiolus* | 3 | 5.13 | 0.18 | 4.98 | 5.32 | 0.34 |
| *Heliophorus moorei* | 3 | 11.09 | 0.26 | 10.78 | 11.26 | 0.48 |
| *Celastrina* sp | 3 | 8.34 | 0.41 | 7.98 | 8.78 | 0.80 |
| *Heliophorus sena* | 3 | 7.08 | 0.23 | 6.90 | 7.34 | 0.44 |
| *Heliophorus* sp | 3 | 5.04 | 0.28 | 4.77 | 5.34 | 0.57 |
| *Cyrestis thyodamas* | 3 | 9.53 | 0.29 | 9.20 | 9.72 | 0.53 |
| *Dodona durga* | 3 | 5.03 | 0.09 | 4.93 | 5.11 | 0.18 |
| *Aglais caschmirensis* | 3 | 0.97 | 0.06 | 0.91 | 1.04 | 0.12 |
| *Coccinella undecimpunctata* | 3 | 0.34 | 0.01 | 0.32 | 0.35 | 0.02 |
| *Coccinella septempunctata* | 3 | 0.83 | 0.08 | 0.74 | 0.88 | 0.14 |

| **Site 5 (2200m) Index of Visitation Rate** | | | | | | |
| --- | --- | --- | --- | --- | --- | --- |
| **Species** | **n** | **mean** | **sd** | **min** | **max** | **Range** |
| *Apis* sp | 3 | 6.44 | 0.38 | 6.13 | 6.86 | 0.73 |
| *Bombus* sp | 3 | 9.14 | 0.12 | 9.01 | 9.23 | 0.23 |
| *Camponotus pennyslyvanicus* | 3 | 5.68 | 0.28 | 5.36 | 5.90 | 0.54 |
| *Eristalis cerealis* | 3 | 36.46 | 1.44 | 35.05 | 37.93 | 2.88 |
| *Eristalis tenax* | 3 | 27.68 | 0.80 | 26.81 | 28.40 | 1.59 |
| *Musca domestica* | 3 | 51.50 | 1.81 | 49.77 | 53.38 | 3.60 |
| *Episyrphus viridaureus* | 3 | 37.67 | 0.73 | 36.83 | 38.17 | 1.33 |
| *Episyrphus balteatus* | 3 | 31.52 | 0.66 | 30.94 | 32.24 | 1.30 |
| *Lucilia* sp | 3 | 24.30 | 0.93 | 23.25 | 25.00 | 1.76 |
| *Syritta* sp | 3 | 3.12 | 0.18 | 2.93 | 3.29 | 0.36 |
| *Celastrina argiolus* | 3 | 9.59 | 0.40 | 9.15 | 9.95 | 0.79 |
| *Heliophorus moorei* | 3 | 6.63 | 0.34 | 6.32 | 6.99 | 0.67 |
| *Heliophorus sena* | 3 | 7.37 | 0.23 | 7.15 | 7.61 | 0.47 |
| *Cyrestis thyodamas* | 3 | 9.06 | 0.53 | 8.67 | 9.66 | 1.00 |
| *Dodona durga* | 3 | 12.07 | 0.25 | 11.79 | 12.30 | 0.50 |
| *Aglais caschmirensis* | 3 | 1.55 | 0.29 | 1.26 | 1.85 | 0.59 |
| *Pieris* sp | 3 | 0.78 | 0.11 | 0.67 | 0.88 | 0.22 |
| *Coccinella septempunctata* | 3 | 1.68 | 0.34 | 1.49 | 2.08 | 0.59 |
| *Coccinella undecimpunctata* | 3 | 0.53 | 0.07 | 0.47 | 0.61 | 0.14 |

|  | **n** | **mean** | **sd** | **min** | **max** | **Range** |
| --- | --- | --- | --- | --- | --- | --- |
| Foraging Behaviour (FB) | 51 | 3.95 | 2.49 | 0.40 | 9.20 | 8.80 |
| Insect Visiting Efficiency (IVE) | 51 | 0.13 | 0.09 | 0.01 | 0.35 | 0.34 |
| Foraging Speed (FS) | 51 | 31.17 | 15.66 | 5.70 | 54.10 | 48.40 |
| Index of visitation rate (IVR) | 51 | 22.15 | 22.90 | 0.34 | 71.07 | 70.73 |

| **Descreptive Statistics by altitude** |
| --- |

**Site-1 (800 m)**

**Site-2 (1150 m)**

|  | **n** | **mean** | **sd** | **min** | **max** | **Range** |
| --- | --- | --- | --- | --- | --- | --- |
| Foraging Behaviour (FB) | 63 | 4.04 | 2.81 | 0.70 | 9.80 | 9.10 |
| Insect Visiting Efficiency (IVE) | 63 | 0.12 | 0.09 | 0.01 | 0.29 | 0.27 |
| Foraging Speed (FS) | 63 | 29.76 | 15.64 | 6.10 | 57.40 | 51.30 |
| Index of visitation rate (IVR) | 63 | 18.50 | 20.21 | 0.72 | 69.63 | 68.91 |

**Site-3 (1500 m)**

|  | **n** | **Mean** | **sd** | **min** | **max** | **Range** |
| --- | --- | --- | --- | --- | --- | --- |
| Foraging Behaviour (FB) | 57 | 4.67 | 2.31 | 0.10 | 9.40 | 9.30 |
| Insect Visiting Efficiency (IVE) | 57 | 0.15 | 0.08 | 0.03 | 0.29 | 0.26 |
| Foraging Speed (FS) | 57 | 31.12 | 12.94 | 6.80 | 50.90 | 44.10 |
| Index of visitation rate (IVR) | 57 | 19.23 | 16.04 | 1.14 | 50.65 | 49.50 |

**Site-4 (1850 m)**

|  | **n** | **Mean** | **sd** | **min** | **max** | **Range** |
| --- | --- | --- | --- | --- | --- | --- |
| Foraging Behaviour (FB) | 66 | 4.04 | 2.48 | 0.20 | 9.90 | 9.70 |
| Insect Visiting Efficiency (IVE) | 66 | 0.12 | 0.09 | 0.02 | 0.27 | 0.25 |
| Foraging Speed (FS) | 66 | 24.81 | 14.22 | 6.10 | 49.10 | 43.00 |
| Index of visitation rate (IVR) | 66 | 11.94 | 9.24 | 0.32 | 37.54 | 37.21 |

**Site-5 (2200 m)**

|  | **n** | **mean** | **sd** | **min** | **max** | **Range** |
| --- | --- | --- | --- | --- | --- | --- |
| Foraging Behaviour (FB) | 57 | 3.87 | 2.49 | 0.30 | 9.90 | 9.60 |
| Insect Visiting Efficiency (IVE) | 57 | 0.11 | 0.10 | 0.01 | 0.30 | 0.29 |
| Foraging Speed (FS) | 57 | 23.94 | 15.74 | 5.20 | 47.40 | 42.20 |
| Index of visitation rate (IVR) | 57 | 14.88 | 14.89 | 0.47 | 53.38 | 52.91 |

| **Descreptive Statistics by altitude** |
| --- |

**Site-1 (800 m)**

|  | **n** | **mean** | **sd** | **min** | **max** | **Range** |
| --- | --- | --- | --- | --- | --- | --- |
| Density | 17 | 1.54 | 1.25 | 0.10 | 3.50 | 3.40 |
| Relative abundance (RA) | 17 | 5.88 | 4.78 | 0.38 | 13.36 | 12.98 |

**Site-2 (1150 m)**

|  | **n** | **mean** | **sd** | **min** | **max** | **Range** |
| --- | --- | --- | --- | --- | --- | --- |
| Density | 21 | 1.61 | 1.33 | 0.30 | 4.20 | 93.9 |
| Relative abundance (RA) | 21 | 4.76 | 3.91 | 0.88 | 12.39 | 11.5 |

**Site-3 (1500 m)**

|  | **n** | **mean** | **sd** | **min** | **max** | **Range** |
| --- | --- | --- | --- | --- | --- | --- |
| Density | 19 | 2.12 | 1.34 | 0.2 | 4.50 | 4.3 |
| Relative abundance (RA) | 19 | 5.26 | 3.33 | 0.5 | 11.19 | 10.7 |

**Site-4 (1850 m)**

|  | **n** | **mean** | **sd** | **min** | **max** | **range** |
| --- | --- | --- | --- | --- | --- | --- |
| Density | 22 | 2.19 | 1.00 | 0.20 | 4.00 | 3.8 |
| Relative abundance (RA) | 22 | 4.49 | 2.05 | 0.41 | 8.21 | 7.8 |

**Site-5 (2200 m)**

|  | **n** | **mean** | **sd** | **min** | **max** | **range** |
| --- | --- | --- | --- | --- | --- | --- |
| Density | 19 | 2.32 | 1.30 | 0.4 | 5.00 | 4.60 |
| Relative abundance (RA) | 19 | 5.23 | 2.92 | 0.9 | 11.26 | 10.36 |
