# Supplementary material for: Plant-pollinator interactions along the altitudinal gradient in Berberis lycium royle: An endangered medicinal plant of the Himalayan region
Source: PLoS One. 2025 May 7;20(5):e0310572. doi: 10.1371/journal.pone.0310572 (PMC12058197; doi:10.1371/journal.pone.0310572)
Supplement: Attachment — Submitted filename: PONE-D-24-38069_reviewed paper.pdf [file pone.0310572.s002.pdf]

PLOS ONE

Plant-Pollinator Interactions along the Altitudinal Gradient in Berberis lycium Royle: An Endangered Medicinal Plant Species of the Himalayan Region

--Manuscript Draft--

Manuscript Number:	PONE-D-24-38069
Article Type:	Research Article
Full Title:	Plant-Pollinator Interactions along the Altitudinal Gradient in Berberis lycium Royle: An Endangered Medicinal Plant Species of the Himalayan Region
Short Title:	Plant-Pollinator Interactions along the Altitudinal Gradient in the Himalaya
Corresponding Author:	Nakul Chettri, PhD<br>International Centre for Integrated Mountain Development<br>Kathmandu, Lalitpur NEPAL
Keywords:	Berberis lycium; pollination; Insect pollinators; Foraging activity; Altitudinal gradients
Abstract:	Mountain ecosystems influence species distribution by offering climatic variables intertwined with rising altitude. These climatic factors determine species phenology and niche width. Although the distributional patterns of some prominent insect groups in relation to altitude have been determined, the environmental preferences along the altitudinal range that differentially influence the pollination of specific plant species are unknown. Here we assess how the composition and abundance of pollinator fauna of the important medicinal plant Berberis lycium Royle (Berberidaceae) differ across five distinct altitudinal gradients (800-2200 m) in the Pir-Panjal mountain range in the northwestern part of the Indian Himalayas. We monitored insect pollinators of major groups (bees, butterflies, wasps, flies) over two consecutive flowering seasons during 2022-2023. In total, 39 insect species belonging to five orders and 17 families were observed visiting the plant species during the flowering period across the altitudinal gradient. The results of the linear regression model depict that all four pollination indices show a negative correlation with increasing altitude in foraging activities when all the data are pooled together. However, only foraging speed (FS) and index of visiting rate (IVR) were statistically significant. In the case of individual orders, only Lepidoptera exhibited a notable relation to altitude. However, asynchrony in foraging activities among other pollinator groups has been reported along altitudinal gradients. The reproductive output (fruit and seed production) shows a significant negative correlation with increasing altitude. We concluded that while altitude influences species distribution, it also differentially shapes plant-pollinator interactions, pollinator activities, and reproductive output. This work is of great significance in order to monitor plant-pollinator interactions, which are essential component of biodiversity rich but fragile mountain ecosystem.
Order of Authors:	Nahila Anjum, PhD
	Sajid Khan, PhD
	Susheel Verm, PhD
	Kailash S. Gaira, PhD
	Balwant Rawat, PhD
	Mohd Hanief, PhD
	Nakul Chettri, PhD
Additional Information:	
Question	Response
Financial Disclosure<br><br>Enter a financial disclosure statement that describes the sources of funding for the	The author(s) received no specific funding for this work.

Powered by Editorial Manager® and ProduXion Manager® from Aries Systems Corporation

work included in this submission. Review the submission guidelines for detailed requirements. View published research articles from *PLOS ONE* for specific examples.<br><br>This statement is required for submission and **will appear in the published article** if the submission is accepted. Please make sure it is accurate.<br><br>**Funded studies**<br>Enter a statement with the following details:<br>• Initials of the authors who received each award<br>• Grant numbers awarded to each author<br>• The full name of each funder<br>• URL of each funder website<br>• Did the sponsors or funders play any role in the study design, data collection and analysis, decision to publish, or preparation of the manuscript?<br><br>Did you receive funding for this work?	
**Competing Interests**<br><br>Use the instructions below to enter a competing interest statement for this submission. On behalf of all authors, disclose any competing interests that could be perceived to bias this work—acknowledging all financial support and any other relevant financial or non-financial competing interests.<br><br>This statement is required for submission and **will appear in the published article** if the submission is accepted. Please make sure it is accurate and that any funding sources listed in your Funding Information later in the submission form are also declared in your Financial Disclosure statement.<br><br>View published research articles from *PLOS ONE* for specific examples.	The authors have declared that no competing interests exist

**NO authors have competing interests**<br><br>Enter: *The authors have declared that no competing interests exist.*<br><br>**Authors with competing interests**<br><br>Enter competing interest details beginning with this statement:<br><br>*I have read the journal's policy and the authors of this manuscript have the following competing interests: [insert competing interests here]*<br><br>* typeset	
**Ethics Statement**<br><br>Enter an ethics statement for this submission. This statement is required if the study involved:<br><br>• Human participants<br>• Human specimens or tissue<br>• Vertebrate animals or cephalopods<br>• Vertebrate embryos or tissues<br>• Field research<br><br>Write "N/A" if the submission does not require an ethics statement.<br><br>General guidance is provided below. Consult the submission guidelines for detailed instructions. **Make sure that all information entered here is included in the Methods section of the manuscript.**	N/A

**Format for specific study types**<br><br>**Human Subject Research (involving human participants and/or tissue)**<br>• Give the name of the institutional review board or ethics committee that approved the study<br>• Include the approval number and/or a statement indicating approval of this research<br>• Indicate the form of consent obtained (written/oral) or the reason that consent was not obtained (e.g. the data were analyzed anonymously)<br><br>**Animal Research (involving vertebrate animals, embryos or tissues)**<br>• Provide the name of the Institutional Animal Care and Use Committee (IACUC) or other relevant ethics board that reviewed the study protocol, and indicate whether they approved this research or granted a formal waiver of ethical approval<br>• Include an approval number if one was obtained<br>• If the study involved *non-human primates*, add *additional details* about animal welfare and steps taken to ameliorate suffering<br>• If anesthesia, euthanasia, or any kind of animal sacrifice is part of the study, include briefly which substances and/or methods were applied<br><br>**Field Research**<br><br>Include the following details if this study involves the collection of plant, animal, or other materials from a natural setting:<br>• Field permit number<br>• Name of the institution or relevant body that granted permission	
**Data Availability**<br><br>Authors are required to make all data underlying the findings described fully available, without restriction, and from the time of publication. PLOS allows rare exceptions to address legal and ethical concerns. See the PLOS Data Policy and FAQ for detailed information.	No - some restrictions will apply

Powered by Editorial Manager® and ProduXion Manager® from Aries Systems Corporation

A Data Availability Statement describing where the data can be found is required at submission. Your answers to this question constitute the Data Availability Statement and **will be published in the article**, if accepted.<br><br>**Important:** Stating 'data available on request from the author' is not sufficient. If your data are only available upon request, select 'No' for the first question and explain your exceptional situation in the text box.<br><br>Do the authors confirm that all data underlying the findings described in their manuscript are fully available without restriction?	
**Describe where the data may be found in full sentences. If you are copying our sample text, replace any instances of XXX with the appropriate details.**<br><br>• If the data are **held or will be held in a public repository**, include URLs, accession numbers or DOIs. If this information will only be available after acceptance, indicate this by ticking the box below. For example: *All XXX files are available from the XXX database (accession number(s) XXX, XXX.*).<br>• If the data are all contained **within the manuscript and/or Supporting Information files**, enter the following: *All relevant data are within the manuscript and its Supporting Information files.*<br>• If neither of these applies but you are able to provide **details of access elsewhere**, with or without limitations, please do so. For example:<br><br>*Data cannot be shared publicly because of [XXX]. Data are available from the XXX Institutional Data Access / Ethics Committee (contact via XXX) for researchers who meet the criteria for access to confidential data.*<br><br>*The data underlying the results presented in the study are available from (include the name of the third party*	The data underlying the results presented in the study are available from the first author on request

and contact information or URL).<br>• This text is appropriate if the data are owned by a third party and authors do not have permission to share the data.<br><br>* typeset	
Additional data availability information:	

Manuscript

Click here to access/download;Manuscript;Plos_One_Manuscript_Main_Rev

Plant-Pollinator Interactions along the Altitudinal Gradient in *Berberis lycium* Royle: An Endangered Medicinal Plant Species of the Himalayan Region

Nahila Anjum[1], Sajid Khan[1], Susheel Verma[2], Kailash S. Gaira[3], Balwant Rawat[4], Mohd Hanief[1], Nakul Chettri[5]*

[1] Department of Botany, School of Biosciences and Biotechnology, Baba Ghulam Shah Badshah University, Rajouri, Jammu and Kashmir, India-185234

[2] Department of Botany, University of Jammu, Jammu and Kashmir, India-180006

[3]G. B. Pant National Institute of Himalayan Environment, Sikkim Regional Centre, Pangthang, Gangtok 737101, Sikkim, India

[4] School of Agriculture, Graphic Era Hill University, Dehradun 248002, Uttarakhand, India

[5]International Centre for Integrated Mountain Development, Post Box 3226, Kathmandu, Nepal

***Correspondence: nakul.chettri@icimod.org**

Abstract

Mountain ecosystems influence species distribution by offering climatic variables intertwined with rising altitude. These climatic factors determine species phenology and niche width. Although the distributional patterns of some prominent insect groups in relation to altitude have been determined, the environmental preferences along the altitudinal range that differentially influence the pollination of specific plant species are unknown. Here we assess how the composition and abundance of pollinator fauna of the important medicinal plant *Berberis lycium* Royle (Berberidaceae) differ across five distinct altitudinal gradients (800-2200 m) in the Pir-Panjal mountain range in the northwestern part of the Indian Himalayas. We monitored insect pollinators of major groups (bees, butterflies, wasps, flies) over two consecutive flowering seasons during 2022-2023. In total, 39 insect species belonging to five orders and 17 families were observed visiting the plant species during the flowering period across the altitudinal gradient. The results of the linear regression model depict that all four pollination indices show a negative correlation with increasing altitude in foraging activities when all the data are pooled together. However, only foraging speed (FS) and index of visiting rate (IVR) were statistically significant. In the case of individual orders, only Lepidoptera exhibited a notable relation to altitude. However, asynchrony in foraging activities among other pollinator groups has been reported along altitudinal gradients. The reproductive output (fruit and seed production) shows a significant negative correlation with increasing altitude. We concluded that while altitude influences species distribution, it also differentially shapes plant-pollinator interactions, pollinator activities, and reproductive output. This work is of great significance in order to monitor plant-pollinator interactions, which are essential component of biodiversity rich but fragile mountain ecosystem.

Keywords: *Berberis lycium*, Pollination, Insect pollinators, Foraging activity, Altitudinal gradients.

1. Introduction

Mountain ecosystems are extremely fragile influenced by numerous drivers on species diversity and distribution patterns. They are home to about half of the world's biodiversity hotspots and nurture numerous endemic species [1]. The Himalaya processes unique bio-climatic geographical ranges in Asia that separate the Tibetan plateau from the lowland plains of the Indian subcontinent. The degree of exposure to weather and environment varies along the different altitudinal zones [2]. Apart from the typical lapse rate in temperature conditions, there is substantial variation in wind speed and direction, orographic patterns, humidity, and precipitation, which differentially affect biodiversity at varying elevations [3].

For both plants and animals, including insects, patterns of species richness, and abundance often show a continually declining trend or a mid-elevation peak in response to elevation, however, these patterns differ between taxa and geographical regions [4-9]). These patterns of abundance and richness are predominantly shaped by abiotic variables physically bound to elevation [4, 10] that result in diversity-limiting factors at higher altitudes. On an average the Himalayas exhibit a standard temperature lapse rate of 5.65°C per 1000 m [11]. As a result, the higher altitude significantly alters the duration of the growth period [12] and metabolic rates [13] in insects. Unsuitable environmental conditions hamper their survival and altitudinal distributional range [14]. As the different potential pollinator insect groups appear to be impacted to varying degrees [15], communities of

pollinators participating in the process of reproduction of mountainous angiospermic plants are likely to vary with elevation gradients [16].

The Pollinator assemblage of insect-pollinated plants remarkably exhibits temporal and spatial variations among plant communities [17] and sometime within a single plant species as well [18]. For example, orchids were pollinated by variable pollinators in different habitats and regions, for different preferences including nectar, pollen, trichomes, fragrance, lipids and resins [19] by a variety of morphologically distinct and specialized pollinators [20]. In addition, pollination is a vital regulating ecosystem service that underpins food production and contributes to gene flow and ecosystem restoration [21, 22]. Eventually the expected far-reaching impacts of the abiotic environmental factors on plant-pollinator interactions, we have little knowledge of how these interactions play out at altitudinal scales. Despite, the fact that several important plant species completely depend upon the insect pollinators for their survival, spatial variation in the pollinator groups along the altitudinal gradient is poorly understood.

Insects are the major pollinators of flowering plants in higher altitudes [23]. ~~These insects~~ are classified into several orders; the most common are Hymenoptera, Lepidoptera, Diptera, Hemiptera and Coleoptera [24]. ~~Many~~ researchers worldwide have studied the insect pollinator spectrum and pollination ecology of numerous medicinally important plants [25]. There are, however, few studies that assess all the necessary variables to ascertain how altitude influences the behaviours and range limits of species, including pollinator visiting rates, abundance, and reproductive output. . Altitudinal gradients provide the best opportunity for observing pollination ecology towards range limits as decline in pollination and resources may limit reproductive output at high altitudes [26, 27].

Berberidaceae is a diverse angiospermic family consisting of 17 genera and 650 species globally, most of which are located in the northern hemisphere. *Berberis lycium* Royle, an extremely useful shrub and most common member of the Berberidaceae family is native to the Himalayan region but may be found worldwide in subtropical and temperate climate. It is an extremely valuable medicinal plant that has been utilised in traditional medicine since time immemorial. Numerous medicinal uses of *B. lycium* were demonstrated, including wound healing, hepatoprotective, antioxidant, antibacterial, antihyperlipidemic and much more. In addition, the plant is traditionally used to treat ophthalmic, liver, skin, cough, and stomach problems. Because of its enormous nutritional and medicinal benefits, it is being overexploited for consumption and trade, which poses a serious threat to the existence of this plant in its natural habitat. The habitat of this plant is also being diminished by other anthropogenic activities like building and road construction, deforestation, agricultural road expansion in hilly areas, and so forth, which is causing a population decline [28, 29]. As per IUCN criteria of 2000, *B. lycium* has already been declared endangered in northwestern region of the Himalayas [30]. Therefore, conservation measures need to be taken to protect this species from additional threat in the near future.

In the northwestern Himalayas, *B. lycium* grows along a altitudinal gradient from 800 to 2200 m asl. Earlier, some studies [31] ~~showed how~~ climatic conditions could influence floral phenology and frequency, activity and life cycle of insect pollinators of a *Berberis* species. Owing to the wide range of habitat distribution of *B. lycium* and reproductive dependence of its insect pollinators [25, 30]. The present study is aimed to (1) document the diversity and variations of prominent insect pollinators of a common host plant species along

altitudinal gradient; (2) compare the insect activities vary along altitudinal gradient; (3) evaluate how diversity and relative abundance of insect visitors influence reproductive output of *B. lycium* along altitudinal gradient. We discussed and suggested possible hypotheses that explain the pollination patterns observed as well as their implications in the context of the conservation of this substantially important medicinal plant.

2. Materials and Methods

2.1. Study area and flowering phenology

Figure 1. here

The present study was conducted at five different altitudes (800m, 1150m, 1500m 1850m, and 2200m; 33°32'-33°58'N and 74°32'-74°41'E) of Pir-Panjal mountain range in Jammu and Kashmir of north western Indian Himalaya from January, 2022 to August 2023. The Pir-Panjal mountain range splits up the Kashmir valley from the plains of the Jammu division (**Figure 1**). The largest mountain range in the lesser Himalayas is the Pir-Panjal range with varying average elevations from 1400 to 4100 m asl [2]. This altitudinal range offers the mountains and hillocks surrounding the Kashmir valley, and has enriched biodiversity above the elevation of 1700 m asl. The objective of this study embraces an diverse range of habitats which is reflected by a distinctive range of biodiversity distribution. The flowering and fruiting season of the plant extends from January–July. The flowers start appearing in January and continue up to June. The ripening of fruit begins in May and becomes fully matured up to early August at study sites along altitudinal gradients.

2.2. Sampling and activities of insect pollinators

Documentation and monitoring of insect pollinators were conducted at the study sites between 08:00 h to 18:00 h from January to June when *B. lycium* was in full bloom for two consecutive flowering seasons during 2022-2023. In *B. lycium*, the efficiency of pollination by insects was monitored such as the category of insects visiting the plant, their foraging behavior, and the flower handling duration. To study the role that insects play in pollination, we used the insect net to catch notable flower visitor. Then we observed them under a stereozoom to examine the load of pollen on different parts of their body. For 30 plants of *B. lycium* in each site, three hours at various times of the day were devoted to observation and capturing the insect visitors to each plant. The specimens of the insect pollinators of *B. lycium* were identified at ITL (Insect Taxonomy Lab.), Department of Zoology, Baba Ghulam Shah Badshah University Rajouri. On each plant, we monitored 15-30 inflorescences, and total insect visitors in the observed inflorescences on 10 plants were considered in each site. During the peak flowering time,(mid-February up to early May) visiting insects were recorded at regular intervals of one hour throughout a 24-hour duration. To observe nocturnal foragers on *B. lycium*, a battery-operated torch was used. Due to the lack of nocturnal foragers on the plant, observations were limited from 0600 to 2000 hours (overall observation period =545 h).

The contribution of each pollinator to pollen transport was determined using various pollination indices:

2.2.1. Foraging behaviour (FB): It was calculated as the time spent by a specific pollinator per visit per inflorescence, counted by a stopwatch [32].

2.2.2. Foraging speed (FS): The number of flowers visited per minute by a pollinator is represented by FS. It was calculated as per [33]:

$$FS = (Fi/di) \times 60$$

where 'di' is the time(s) shown by the stopwatch, and 'Fi' is the number of flowers visited during 'di'.

2.2.3. Index of visitation rate (IVR): IVR is a relative measure of visitor rate that takes into consideration both activity rate and frequency of visits. It was calculated by following earlier work [32] as follows,

$$IVR = F \times AR$$

Where, 'F' represents the proportion of visiting-insect individuals to the total number of insects in the census, whereas 'AR' stands for activity rate, or the average number of flowers visited by a visiting-insect category in a minute.

2.2.4. Insect visiting efficiency (IVE): IVE was calculated following [32],

$$IVE = \frac{\text{Number of total flowers visited by insects in one visit}}{\text{Number of total flowers available}}$$

2.2.5. Potential insect pollinator: To determine potential pollinators of *B. lycium,* the behaviour of insects was examined at two distinct behavioural decision levels.

(i) Relative Abundance: The number of insect pollinators on *B. lycium* was observed on 15-30 inflorescences in each plant on a total of 30 plants in each site. The low or high abundance specified accessory, and major pollinators, respectively.

(ii) Sought floral resource: Insects were grouped on the basis of their purpose of visit on *B. lycium* like Collecting Pollen (CP), and Feeding on Pollen (FP), and Feeding on Nectar (FN).

2.3. Reproductive output

To calculate the reproductive output, the percentage of fruit and seed set by each plant was recorded. Percentage fruit set was determined by calculating the average number of flowers, and fruits per plant (Verma et al., 2021)[30]. Percentage fruit set was determined by using the formula:

$$\text{Percentage fruit set} = \frac{\text{Average number of fruit formed per plant}}{\text{Average number of flowers produced per plant}} \times 100$$

For the determination of percentage seed set, average number of ovules per ovary, and, average number of flower per plant was calculated [30]. Percentage seed set was calculated by using the formula:

$$\text{Percentage seed set} = \frac{\text{Average number of seeds per fruit}}{\text{Average number of ovules per ovary}} \times 100$$

2.4. Data analysis

All the statistical analyses were carried out using R software version 4.0.2 (R Core Team 2020)[34]. Before data analysis, we evaluated whether the data on all studied pollination indices (foraging speed, foraging behavior, index of visitation rate, insect visiting efficiency, and relative abundance) met the requirement of homogeneity of variance and normality of distribution by using Levene's test, and the Shapiro-Wilk normality test, respectively [35]. Since the assumptions of homogeneity of variance, and normality was not met for the studied pollination indices ($p<0.001$), we performed simple linear regression to study how insect activity and relative abundance as a function of altitude for all the order-wise data pooled together. Additionally, basic linear regression was used to examine the relationship between insect activity and pollen density. We checked the homogeneity and normality assumptions of the data using Levene's test and the Shapiro-Wilk test before analyzing the variation in the insect pollination indices along the altitudinal gradient. In order to determine if there is a significant difference in the pollination indices along the altitudinal gradient, we used the unpaired two-sample T-test because the necessary assumptions have been met in the data ($p>0.05$).

3. Results

3.1. Plant species, floral arrangement and pollination

B. lycium is an evergreen, spiny, large shrub that grows at an altitudinal range between 800–2200 m asl. The leaves are coriaceous, slightly obovate-oblong or lanceolate having a few large, spiny teeth arranged alternately along the stem. The inflorescence is corymbose racemes containing 18-24 bright yellow, bracteate, hypogynous, actinomorphic, and hermaphrodite flowers that grow in axillary clusters. Over the calyx, two sporophylls are present. Six light yellow-colored sepals are present in two whorls, the outer three are smaller than the inner three. The corolla has six petals and is bright yellow. At the base of each petal, two nectaries are present. The androecium is composed of six antipetalous, adnate, and bithecous stamens. A single pistil is divided into the style, stigma, and ovary representing the gynoecium. The stigma has a central depression and is spherical. Style is short and empty. Pollination in *B. lycium*is carried out by insects as the plant possesses all the necessary entomophilous components, including brighter colour, scent, nectar, and pollen. Insect interaction is necessary for the pollen deposition on the stigmatic surface. The fruits are oval-shaped berries; acquire bright red or purple colour when fully ripened. Fruits contain 2-5 seeds which are yellow to pink in colour.

3.2. Species composition and activities of insect pollinators

In total, 1,934 individuals belonging to five orders, 17 families, and 39 species were found visiting *B. lycium* during the flowering period across altitudinal gradients. At site-1 (800 m), Diptera was the most dominant order with 8 species, followed by the Hymenoptera with four species, Lepidoptera, and Hemiptera each with 2 species, and Coleoptera with single species. At site-2 (1150 m), again Diptera was the most dominant order with 11 species, followed by Hymenoptera with 4 species, Hemiptera, Lepidoptera, and Coleoptera each having 2 species. At site-3 (1500 m), Diptera was the most dominant order again with 11 species, followed by Hymenoptera and Lepidoptera each with 3 species, and Hemiptera and Coleoptera each consisting of single species. However, at site-4 (1850 m), Lepidoptera was the most dominating order with 8 species followed by Diptera with 7 species, Hymenoptera with 5 species and, Coleoptera with 2 species. At site-5 (2200 m), Lepidoptera and Hymenoptera

both consisting of 7 species followed by Hymenoptera with 3 species and Coleoptera with 2 species (Table 1 and Figure 2 and 3).

Figure 2. Here.

**Species**	**Order**	**Family**	**Foraging activity**	**Altitude (m)**
Allograpta sp.	Hymenoptera	Apidae	FN	800
Apis sp.	Hymenoptera	Apidae	FN, CP, FP	800-2200
Bombus trifasciatus	Hymenoptera	Apidae	FN	800-1150
Formica fusca	Hymenoptera	Formicidae	FP	800-1150
Bombus sp.	Hymenoptera	Apidae	FN, CP	1850-2200
Camponotuspennyslyvanicus	Hymenoptera	Formicidae	FN, CP, FN	1500-2200
Bombus haemorrhoidalis	Hymenoptera	Apidae	FN, CP	1850
Apis cerena	Hymenoptera	Apidae	FN, CP, FP	1500-1850
Coelioxys sp.	Hymenoptera	Megachilidae	FN, CP	800
Episyrphusbalteatus	Diptera	Syrphidae	FP	800-2200
Eristalistenax	Diptera	Syrphidae	FP	800-2200
Musca domestica	Diptera	Muscidae	FP	800-2200
Calliphora vomitoria	Diptera	Calliphoridae	FN, CP, FP	800-1150
Chrysotoxumbaphyrum	Diptera	Syrphidae	FN	800-1150
Eupeodesluniger	Diptera	Syrphidae	FP	800-1500
Sarchophaga sp.	Diptera	Sarchophagidae	FP	800-1500
Plecia sp.	Diptera	Bibionidae	FP	800-1150
*Rhingia*sp.	Diptera	Syrphidae	FN, FP	1150-1500
*Calliphora*sp	Diptera	Calliphoridae	FN, CP, FP	1150
Eristaliscerealis	Diptera	Syrphidae	FP	1150-2200
Episyrphysviridaureus	Diptera	Syrphidae	FP	1500-2200
Eristalinustaeniops	Diptera	Syrphidae	FP	1550
Lucilia sp.	Diptera	Calliphoridae	FN, CP, FN	1500-2200
Syritta sp.	Diptera	Syrphidae	FP	1500-2200
*Dyscedrus*sp.	Hemiptera	Coreidae	FP	800-1150
Aphidoidea	Hemiptera	Aphididae	FP	800-1500
Praezygaenacaschmirensis	Lepidoptera	Zygaenidae	CP, FP	800-1500
Celastrinaargiolus	Lepidoptera	Lycaenidae	FN	800-2200
Dodona durga	Lepidoptera	Riodinidae	FN	1500-2200
Heliophorusmoorei	Lepidoptera	Lycaenidae	FN	1850-2200
Celastrina sp.	Lepidoptera	Lycaenidae	FN	1850

Heliophorus sena	Lepidoptera	Lycaenidae	FN	1850-2200
*Heliophorus*sp.	Lepidoptera	Lycaenidae	FN	1850
Cyrestisthyodamas	Lepidoptera	Nymphalidae	FN	1850-2200
Aglaiscaschmirensis	Lepidoptera	Nymphalidae	FN	1850-2200
Pieris sp.	Lepidoptera	Pieridae	FN	2200
Coccinellaundecimpunctata	Coleoptera	Coccinellidae	CP	800-2200
Altica sp.	Coleoptera	Chrysomelidae	FN, FP	1150-1500
Coccinella septempunctata	Coleoptera	Coccinellidae	CP	1850-2200

Table 1. Showing insect pollinator species belonging to different orders, their foraging activity and altitudinal range (FN= Feeding on Nectar, CP= Collecting Pollen, and FP=Feeding on Pollen).

Figure 3. here

3.3. Insect activities:

The results of the linear regression showed a statistically non-significant increase (*p*>0.05) in terms of foraging behaviour of all the studied insect pollinator orders with the highest increase in Diptera and Hymenoptera with an increase in altitude respectively (Figure 4A).

We found a statistically significant decrease (*p*<0.0001) in the foraging speed of the order Lepidoptera only (y=36.4-0.0111x, R^2 =0.05). However, a statistically non-significant increase (*p*>0.05) in Hymenoptera and Hemiptera and a non-significant decrease (*p*>0.05) in Coleoptera and Diptera have been observed in the studied insect orders with an increase in altitude (Figure 4B).

Figure 4. Here

Further, with an increase in altitude, a statistically significant decrease (*p*<0.05) in insect visitation rate has been observed for the order Diptera (y=38.9-0.00677x, R^2=0.03), whereas, a statistically significant increase (*p*<0.05) for insect visitation rate has been observed for the order Lepidoptera (y=1.96+0.00214x, R^2=0.08). However, a statistically non-significant decrease (*p*>0.05) in terms of IVR has been observed for Coleoptera, Hemiptera, and Hymenoptera insect orders (Figure 4C).

Similarly, a statistically significant increase (*p*<0.05) in insect visiting efficiency was observed for the order Hemiptera (y=0.0155+3.96×10^{-5}x, R^2=0.31) with an increase in altitude. Whereas, a statistically significant decrease (*p*<0.001) has been observed for the order Lepidoptera (y=0.0941-2.37×10^{-5}x, R^2=0.21). However, a statistically non-significant decrease (*p*>0.05) was observed for Coleoptera, and the non-significant increase was observed for Diptera and Hemiptera insect orders with an increase in altitude (Figure 4D).

We found a statistically significant decrease (p<0.005) for foraging speed (y=36.6-0.00566x, R^2=0.03) and index of visitation rate (y=26.4-0.0061x, R^2=0.03) in overall insect species with an increase in attitude. However, a statistically non-significant decrease (p<0.05) was observed in the foraging behaviour and insect visiting efficiency with an increase in altitude (Figure 5).

Figure 5. here.

Then, we found a statistically significant increase (p<0.05) in relative abundance in the case of Lepidoptera (y=0.929+0.00264x, R^2=0.31). However, a statistically non-significant decrease (p<0.05) was observed in Diptera and Hemiptera and a non-significant increase in Coleoptera and Hymenoptera was observed with an increase in altitude (Figure 6).

Figure 6. here

The average percentage fruit set and percentage set decreased with increasing elevation (Figure 7), and the decrease was statistically non-significant (p<0.05) in the case of the percentage fruit set (y=71-0.0154 x, R^2=0.43) and, percentage seed set (80.9-0.0104 x, R^2=0.42) with an increase in altitude.

Figure 7. Here

4. Discussions

In this study, we documented various types of insect pollinators along altitudinal gradients commonly found foraging on flowers of *B. lycium*. In total 1,934 individuals of insect pollinators belong to 39 species under five orders and 17 families were observed visiting the plant species during the flowering period across altitudinal gradient. Pooled insect order data in each altitude were used to assess differences in insect activities and abundance across elevation zones. We observed significant variations in the insect pollination indices along five altitudinal gradients. Our results also indicated differences in the relative abundance of insect pollinators and reproductive output along the altitudinal gradients. However, the repetition of collections in various sampling days in the individual sites offers a broad assessment of species flowering phenology and insect visitors along altitudinal gradients with greater precision.

This is the first study to document potential insect pollinators of *B. lycium* along the altitudinal gradient in the Pir-Panjal region of Indian western Himalaya. We found that *B. lycium* has a more diverse assemblage of pollinators as previously reported [25, 30] due to a wide range of altitudinal gradients. In the case of foraging behavior (FB), the *Apis*species is the most common forager reported in all the studied altitudes (Table 1). However, *Musca domestica, Calliphora vomitoria, Sarchophaga*sp. and *Lucilia* sp. were reported to have the highest foraging behaviour among Hymenoptera. The foraging behaviour of all the studied insect pollinator orders shows a statistically non-significant increase with the highest increase in Diptera and Hymenoptera with an increase in altitude respectively (Figure 4A). In case of foraging speed (FS), *Calliphora vomitoria, Bombus trifasciatus, Sarchophaga*sp. and,*Eupeodesluniger*have the highest foraging speed. However, among all the studied orders a statistically significant decrease in foraging speed was observed in Lepidoptera only (Figure 4B). In the case of Insect visiting efficiency (IVE), *Bombus trifasciatus, Calliphora vomitoria,*and

*Episyrphusbalteatus*showthe highest activity. When data were polled together, only Diptera showed a significant decrease and Lepidoptera shows significant increase in the insect visiting efficiency (IVE) with the increase in altitude respectively (Figure 4C). Further, in index of visitation rate (IVR), *Calliphora vomitoria, Eupeodesluniger, Episyrphusbalteatus,* and *Eristalistenax*shows highest activity. When data polled together, only Lepidoptera shows significant decrease in the (IVR) with the increase in the altitude (Figure 4D). While considering all the four parameters (FB, FS, IVE and, IVR) and data pooled together, the activity rate shows a decrease with an increase in altitude. However, only foraging speed (FS) and index of visitation rate (IVR) show statistically significant results (Figure 5). Lastly, in the case of relative abundance of insect pollinators, Lepidoptera shows a statistically significant increase with the increase in altitude (Figure 6). In the final important observation, we observed a statistically significant decrease in fruit and seed set along the altitudinal gradient (Figure 7).

Observations on major pollinator groups (Hymenoptera, Diptera, Coleoptera, Lepidoptera) may serve as efficient indicators of changes in micro-climate that directly affect their foraging and nesting place [36]. The majority of Pollinators belonging to Lepidoptera show a distinct response to altitude in which they showed their presence in the elevation range between 1500-2200m with few exceptions which signifies its importance in climate change studies [37]. The members of Hemiptera were confined to lower altitudes only; hence they were not considered as potential pollinators of *B. lycium*. The members of Hymenoptera and Diptera showed their presence in a wide range of altitudes. At the order level and, at the base altitude of 800 m, flies (Diptera) predominate (with few exceptions) as potential visitors of *B. lycium* (Table 1). Above 800 m the most prominent flower visiting orders altered among sites with no consistent altitudinal pattern except Lepidoptera. Flies (Diptera) therefore represented comparatively diverse and abundant potential visitors that provide pollination services to *Berberis* to a greater extent [25, 30, 31, 38], and decrease in their abundance and activity along altitudinal gradient can be linked to a decrease in the reproductive output of this essentially important medicinal plant.

Altitudinal variations in insect taxonomic diversity can be observed in varied spatial scales [39]. On a finer scale, the number of insect pollinators associated with a specific plant species is reported to decrease with increasing altitude [6, 8, 40, 41] reported a decline in the insect fauna of *Betula pubescens* (Heteroptera, Coleoptera, and Homoptera species) from 0 to 900 m in Norway. Another study conducted on *Campanula rotundifolia* showed comparatively lower pollinator diversity and visitation rate of insect visitors at higher elevations as highly efficient bumblebees replace less efficient solitary bees in higher elevations [42]. These previous observations are in parallel with our findings as all observed activities of insect visitors show a decrease with increasing altitude. However, only foraging speed and index of visitation rate show statistically significant results (Figure5). Our results indicated that the foraging behaviour of visiting insects of all studied orders increases with increasing altitude (Figure 4A). This variation in foraging behaviour also reflects host plant abundance and richness [14] and also due to the decline in interacting partners at high elevations (Adedoja et al., 2018)[43]. Butterflies and moths are diverse, the most studied insects and highly sensitive to climate, therefore become ideal for climate change research [37]. This coincides with our findings because only Lepidoptera shows a significant decrease in foraging speed with an increase in altitude (Figure 4B). Our work (supplementary table S1) coincides with the previous findings [44], which observed a positive correlation between the diversity of Bumblebees with increasing altitude and a negative correlation between the diversity and visitation rate of non-bumblebees with increasing altitude. In the case of insect visiting efficiency, only Lepidoptera and Hemiptera show significant

decrease and increase in activity respectively (Figure 4D). At higher altitudes of temperate regions, anthophilous flies are often the sole pollinators [45-47] but at lower altitudes presence of Hymenoptera increases. Hemiptera, in our study, plays a minor role and tends to decrease with increasing altitude.

A great diversity of insects are specialized for feeding on host plant reproductive and floral structures [32, 48, 49], but many plant species experience a declined reproductive output with increasing elevation [6, 27, 50, 51]. The fitness of plant-pollinator interactions is essential for plant reproductive assurance and the evolution of floral traits and breeding systems [35]. Breeding systems usually vary within or among different plant lineages that include complete as well as intermediate outcrossers, and selfers [52, 53] and lineages undergo repeated selfing may often suffer the loss of fitness due to inbreeding depression. Therefore, insect pollination has been proven to be a key factor for both the nutritional and, physical quality [54]. Although biotic interactions play a significant role in limiting the expansion of many plant species, pollination syndrome also plays a significant evolutionary function in maintaining ecological range boundaries [55]. Insect pollinator decline is likely to affect plant reproductive traits that grow in vulnerable habitats. For instance, early flowering, and self-incompatible plants are more reliant on pollinators than mid-flowering, and self-compatible plants, therefore pollinator decline is expected to have a significant impact on these types of plants. The high reliance of *B. lycium* on pollinators as observed in our study leads us to speculate that the high dependence on pollinators for the production of seed observed in our study suggests that if plants cannot adjust their reproductive strategies to reduce this reliance amidst pollinator declines, their seed production will be drastically affected by climate change [56].

5. Conclusion

For conservation biologists looking at how plant-pollinator interactions may be affected, our findings could be helpful as global climate change is predicted to alter spatio-temporal distribution and interactions of anthophilous insects. Butterflies and moths (Lepidoptera) appear to be the most sensitive pollinators and show significant variations in their activity along altitudinal gradients. We documented the diversity of anthophilous insects associated with the pollination of *B. lycium* along altitudinal gradients. As a global trend, our study also observed the decrease in the overall abundance and activities of insect pollinators of *B. lycium* with an increase in altitude have a negative correlation with its reproductive outp[illegible]he high dependence of *B. lycium* on pollinators, as observed in our study, suggests that if plants cannot adjust their reproductive strategies to reduce this reliance amid pollinator declines, their seed production will be significantly impacted by climate change.

Supporting information

Supplementary table S1. Showing Foraging Behaviour, Foraging Speed, Insect Visiting Efficiency, Index of Visitation Rate, Density and Relative abundance of various individual insect species and orders at different altitudes.

Author Contributions

Conceptualization: Nahila Anjum, Susheel Verma, Mohd Hanief

Data curation: Nahila Anjum

Formal analysis: Nahila Anjum, Sajid Khan

Funding acquisition: Nahila Anjum

Investigation: Susheel Verma, Kailash S Gaira, Balwant Rawat, Nakul Chettri, Mohd Hanief

Methodology: Nahila Anjum, Sajid Khan

Project administration: Susheel Verma, Nakul Chettri, Mohd Hanief

Resources: Nahila Anjum, Susheel Verma, Mohd Hanief

Software: Nahila Anjum, Sajid Khan

Supervision: Nakul Chettri, Mohd Hanief

Validation: Susheel Verma, Kailash S Gaira, Balwant Rawat, Nakul Chettri, Mohd Hanief

Visualization: Balwant Rawat

Writing – original draft: Nahila Anjum, Sajid Khan

Writing – review & editing: Nahila Anjum, Sajid Khan, Susheel Verma, Kailash S Gaira, Balwant Rawat, Nakul Chettri, Mohd Hanief

Declaration of Competing Interest

The authors declare no conflict of interest.

Acknowledgments

The authors thank Ms. Taslima Sheikh and Dr. Sajad H. Parey, Department of Zoology, BGSB University, Rajouri for insect identification. Besides, first author (Ms. Nahila Anjum) is thankful to CSIR-HRDG, New Delhi for providing Junior and Senior Research Fellowship (File no. 09/1172(0005)/2019-EMR-I).

References

01. Payne D, Spehn EM, Prescott GW, Geschke J, Snethlage MA, Fischer M. Mountain biodiversity is central to sustainable development in the mountains and beyond. One Earth. 2020; 3(5): 530-533.https://doi.org/10.1016/j.oneear.2020.10.013.

02. Khan S, Wani ZA, Ahmad R, Gaira KS, Verma S. Time series analysis of climatic variability and trends in Shiwalik to Pir Panjal mountain range in the Indian western Himalaya. Environ. Devel. Sustain. 2023a; 1-23.https://doi.org/10.1007/s10668-023-03476-7.

03. Dad JM, Muslim M, Rashid I, Reshi ZA. Time series analysis of climate variability and trends in Kashmir Himalaya. Ecol. Indic. 2021; 126: 107690. https://doi.org/10.1016/j.ecolind.2021.107690.

04. Cristobal-Perez EJ, Barrantes G, Cascante-Marín A, Hanson P, Picado B, Gamboa-Barrantes N, Rojas-Malavasi, G, Zumbado MA, Madrigal-Brenes R, Martén-Rodriguez S, Quesada M. Elevational and seasonal patterns of plant pollinator networks in two highland tropical ecosystems in Costa Rica. PloSOne. 2024; 19(1): e0295258. https://doi.org/10.1371/journal.pone.0295258.

05. Wani BA, Wani SA, Magray JA, Ahmad R, Ganie AH, Nawchoo IA. Habitat suitability, range dynamics, and threat assessment of *Swertia petiolata* D. Don: a Himalayan endemic medicinally important plant under climate change. Environ. Monit. Assess. 2023; 195 (1): 214. https://doi.org/10.1007/s10661-022-10773-7.

06. Chen A, Li Z, Zheng Y, Zhan J, Yang B, Yang Z. Decreasing Species Richness with Increase in Elevation and Positive Rapoport Effects of Crambidae (Lepidoptera) on Mount Taibai. Insects 2022; 13 (12): 1125. https://doi.org/10.3390/insects13121125.

07. Dyola N, Sigdel SR, Liang E, Babst F, Camarero JJ, Aryal S, ... & Peñuelas J. Species richness is a strong driver of forest biomass along broad bioclimatic gradients in the Himalayas. *Ecosphere*, 2022 13(6), e4107.https://doi.org/10.1002/ecs2.4107

08. Lefebvre V, Villemant C, Fontaine C, Daugeron C. Altitudinal, temporal and trophic partitioning of flower-visitors in Alpine communities. Sci. Rep. 2018; 8(1): 4706.https://doi.org/10.1038/s41598-018-23210-y.

09. Sundqvist MK, Sanders NJ, Wardle DA. Community and ecosystem responses to elevational gradients: processes, mechanisms, and insights for global change. Annu. Rev.Ecol.Evol.Syst. 2013; 44: 261-280. https://doi.org/10.1146/annurev-ecolsys-110512-135750.

10. Sigdel SR, Liang E, Rokaya MB, Rai S, Dyola N, Sun J. ... & Peñuelas J. (2023). Functional traits of a plant species fingerprint ecosystem productivity along broad elevational gradients in the Himalayas. *Func. Ecol.* 2023; *37*(2), 383-394.https://doi.org/10.1111/1365-2435.14226

11. Nayava JL, Adhikary S, Bajracharya OR. Spatial and temporal variation of surface air temperature at different altitude zone in recent 30 years over Nepal. Mausam 2017; 68(3): 417-428. https://doi.org/10.54302/mausam.v68i3.649.

12. Smith JM, Telemeco RS, Briones Ortiz BA, Nufio CR, BuckleyLB, 2021. High-elevation populations of montane grasshoppers exhibit greater developmental plasticity in response to seasonal cues. Front. Physiol. 2021; 12: 738992. https://doi.org/10.3389/fphys.2021.738992.

13. Koyama T, Texada MJ, Halberg KA, Rewitz K. Metabolism and growth adaptation to environmental conditions in Drosophila. Cell. Mol. Life Sci. 2020; 77(22): 4523-4551. https://doi.org/10.1007/s00018-020-03547-2.

14. Hodkinson ID. Terrestrial insects along elevation gradients: species and community responses to altitude. Biol. Rev. 2005; 80(3): 489-513.https://doi.org/10.1017/S1464793105006767.

15. Ceia-Hasse A, Boieiro M, Soares A, Antunes S, Figueiredo H, Rego C, Borges PA, Conde J, Serrano AR. Drivers of Insect Community Change along the Margins of Mountain Streams in Serra da Estrela Natural Park (Portugal). Insects 2023; 14(3): 243.https://doi.org/10.3390/insects14030243.

16. Xu YW, Sun L, Ma R, Gao YQ, Sun H, Song B. Does pollinator dependence decrease along elevational gradients? Plant Divers. 2023; 45(4): 446-455.https://doi.org/10.1016/j.pld.2023.03.006.

17. Abdusalam A, Liao WJ, Zhang ZQ, Li QJ. Pollinator shifts along an elevation gradient mediate different response in self-pollination in heterostylous *Primula nivalis*. J. Syst. Evol. 2022; 60(1):186-195.https://doi.org/10.1111/jse.12639.

18. Zych M, Junker RR, Nepi M, Stpiczyńska M, Stolarska B, Roguz K. Spatiotemporal variation in the pollination systems of a supergeneralist plant: is A*ngelica sylvestris* (Apiaceae) locally adapted to its most effective pollinators? Ann. Bot.2019; 123(2): 415-428. https://doi.org/10.1093/aob/mcy140.

19. Ackerman JD, Phillips RD, Tremblay RL, Karremans A, Reiter N, Peter CI, Bogarin D, Perez-Escobar OA, Liu H Beyond the various contrivances by which orchids are pollinated: global patterns in orchid pollination biology.Bot. J. Linn. Soc. 2023; 202(3): 295-324. https://doi.org/10.1093/botlinnean/boac082.

20. BobergE, Alexandersson R, Jonsson M, Maa, J, Agren J, Nilsson LA. Pollinator shifts and the evolution of spur length in the moth-pollinated orchid *Platanthera bifolia*. Ann.Bot. 2014; 113(2): 267-275.https://doi.org/10.1093/aob/mct217.

21. Gaira KS, Rawal RS, Singh KK. Variations in pollinator density and impacts on large cardamom (*Amomum subulatum*Roxb.) crop yield in Sikkim Himalaya, India. J.Asia-Pac.Biodivers. 2016; 9: 17e21.https://doi.org/10.1016/j.japb.2015.12.010.

22. Kearns, CA, Inouye DW, Waser NM. Endangered mutualisms: the conservation of plant-pollinator interactions. Annu. Rev. Ecol. Syst. 1998; 29: 83-112. https://doi.org/10.1146/annurev.ecolsys.29.1.83.

23. Li KQ, Ren ZX, Li Q. Diversity of flower visiting beetles at higher elevations on the Yulong Snow Mountain (Yunnan, China). Divers. 2021; 13(11): 604.https://doi.org/10.3390/d13110604.

24. Minachilis K, Kantsa A, Devalez J, Vujic A, Pauly A, Petanidou T. High species turnover and unique plant–pollinator interactions make a hyperdiverse mountain. J. Anim.Ecol. 2023; 92(5): 1001-1015. https://doi.org/10.1111/1365-2656.13898.

25. Badoni A, Arya MK. Insects visiting the medicinal plants from genus *Berberis* L. in the Western Himalaya. Orient. Insects 2022; 56(1): 1-13. https://doi.org/10.1080/00305316.2020.1860148.

26. Hargreaves AL, Weiner JL, Eckert CG. High-elevation range limit of an annual herb is neither caused nor reinforced by declining pollinator service. J. Ecol. 2015; 103 (3): 572-584. https://doi.org/10.1111/1365-2745.12377.

27. Dawson-Glass E, Hargreaves AL. Does pollen limitation limit plant ranges? Evidence and implications. Philos. Trans. R. Soc. Lond. B. Biol. Sci. 2022 14;377(1846):20210014. https://doi.org/10.1098/rstb.2021.0014

28. Anjum N, Ridwan Q, Akhter F, Hanief M. Phytochemistry and therapeutic potential of *Berberis lycium* Royle; an endangered species of Himalayan region. Acta EcologicaSinica 2022; 43 (4): 577-584. https://doi.org/10.1016/j.chnaes.2022.09.005.

29. Anjum N, Ridwan Q, Akhter F, Hanief M. Predicting current and future distribution pattern of *Berberis lycium* Royle concerning climate change using an ensemble modelling approach. Geol. Ecol.Landsc. 2023; 8 (2): 1-10. https://doi.org/10.1080/24749508.2023.2287293.

30. Verma S, Wani IA, Khan S, SharmaS, Kumari P, Kaushik P, El-Serehy HA. Reproductive biology and pollination ecology of *Berberis lycium* Royle: A highly valued shrub of immense medicinal significance. Plants 2021; 10(9): 1907. https://doi.org/10.3390/plants10091907.

31. Radice S, Giordani E, Arena ME. Effect of Climatic Variations in the Floral Phenology of *Berberis microphylla* and Its Pollinator Insects. Hortic. 2023; 9 (12): 1254. https://doi.org/10.3390/horticulturae9121254.

32. Khan S, Kumari P, Verma S. Ambophily in *Olea ferruginea*: a transitional state in the pollination syndrome. Pl. Biosystems 2023b; 157(2): 221-232.https://doi.org/10.1080/11263504.2022.2100497.

33. Pando JB, Fohouo FNT, Tamesse JL. Foraging and pollination behaviour of *Xylocopa calens*Lepeletier (Hymenoptera: Apidae) on *Phaseolus coccineus* L. (Fabaceae) flowers at Yaounde (Cameroon). Entomol. Res. 2011; 41(5): 185-193. https://doi.org/10.1111/j.1748-5967.2011.00334.x.

34. R Core Team (2020) 'R: a language and environment for statistical computing.' (R Foundation for Statistical Computing: Vienna, Austria) Available at https://www.R-project.org/

35. Khan S, Verma S. Extra-stigmatic pollen germination and pistil elongation: a novel strategy towards reproductive assurance in *Dodonaea viscosa* subsp. *angustifolia*. Aust. J. Bot. 2024; 72 (2). https://doi.org/10.1071/BT22062.

36. Chowdhury S, Dubey VK, Choudhury S, Das A, JeengarD, Sujatha B, Kumar A, Kumar N, Semwal A, Kumar V. Insects as bioindicator: A hidden gem for environmental monitoring. Front. Environ. Sci. 2023; 11: 1-16.https://doi.org/10.3389/fenvs.2023.1146052.

37. Hill GM, Kawahara AY, Daniels JC, Bateman CC, Scheffers BR. Climate change effects on animal ecology: butterflies and moths as a case study. Biol. Rev. 2021; 96(5): 2113-2126. https://doi.org/10.1111/brv.12746.

38. Arya MK, Badoni A. *Berberis aristata*DC: An Important Winter Foraging Source for Anthophilous Insects in Western Himalaya. J. Adv. Zool.2023; 44(2): 10–18. https://doi.org/10.17762/jaz.v44i2.64.

39. Masviken J, Marquina D, Noren K, Dalen L, DalerumF. Elevational variation of spider and insect communities in the Swedish mountains. Ecosphere. 2023; 14 (6): e4540.https://doi.org/10.1002/ecs2.4540.

40. Amin M, Mahmood K, BodlahI, QasimM. Assessing diversity of aphids (Hemiptera: Aphididae) along elevation gradient in Azad Jammu and Kashmir-Pakistan. Int. J. Trop. Insect Sci. 2022; 42 (1): 573-580.https://doi.org/10.1007/s42690-021-00573-w.

41. Hagvar S. Altitudinal zonation of the invertebrate fauna on branches of birch (*Betula pubescens*Ehrh). Nor. J.Entomol. 1976;23: 61-74.https://www.cabidigitallibrary.org/doi/full/10.5555/19760538437.

42. Bingham RA, Ranker TA. Genetic diversity in alpine and foothill populations of *Campanula rotundifolia* (Campanulaceae). Int. J. Plant Sci. 2000; 161(3): 403-411. https://doi.org/10.1086/314272.

43. AdedojaOA, Kehinde T, Samways MJ. Insect-flower interaction networks vary among endemic pollinator taxa over an elevation gradient. PloS One 2018; 13(11): e0207453.https://doi.org/10.1371/journal.pone.0207453.

44. Minachilis K, Kantsa A, Petanidou T. Bumblebees, the proficient mountain pollinators: evidence from *Stachys germanica* (Lamiaceae) along the altitudinal gradient of Mount Olympus, Greece. Biol. J. Linn. Soc. 2021; 134 (3): 732-744.https://doi.org/10.1093/biolinnean/blab113.

45. Dellinger AS, Hamilton AM, Wessinger CA, Smith SD. Opposing patterns of altitude-driven pollinator turnover in the tropical and temperate Americas. Am. Nat. 2023; 202(2): 152-165.https://doi.org/10.1086/725017.

46. McCabe LM, Cobb NS. From bees to flies: global shift in pollinator communities along elevation gradients. Front.Ecol.Evol. 2021; 8:626124.https://doi.org/10.3389/fevo.2020.626124.

47. Wotton KR, Gao B, Menz MH, Morris R.K, Ball SG, Lim KS, Reynolds DR, Hu G, Chapman JW. Mass seasonal migrations of hoverflies provide extensive pollination and crop protection services. Curr. Biol. 2019; 29 (13): 2167-2173.https://doi.org/10.1016/j.cub.2019.05.036.

48. Strong WB, Mangini AC, CandauJN. Insects of Reproductive Structures. For.Entomol.Pathol. 2023; 1: 523-579. https://doi.org/10.1007/978-3-031-11553-0-16.

49. Raguso RA. Don't forget the flies: dipteran diversity and its consequences for floral ecology and evolution. Appl. Entomol. Zool. 2020;55**:** 1–7. https://doi.org/10.1007/s13355-020-00668-9.

50. Liao Y, Song X, YeY, Gu J, Wang R, Zhao D, Shao X. Climate Change May Pose Additional Threats to the Endangered Endemic Species *Encalyptabuxbaumioidea*in China. Divers. 2023; 15 (2): 269. https://doi.org/10.3390/d15020269.

51. Singh P, Gargi B, Semwal P, Tripathi V, Global trends, research progress and knowledge mapping of plant–pollinator interactions through bibliometric analysis (1984 to 2023), Biodiversity, 2023 **24**, 4, (200-211). https://doi.org/10.1080/14888386.2023.2258836

52. Khan S, Kumari P, Verma S. Breeding system in *Olea ferruginea* Royle (Oleaceae): comparative performance of staminate and perfect flowers in reproductive assurance. Bot. Lett. 2023c; 171(2): 220–234. https://doi.org/10.1080/23818107.2023.2252888.

53. Whitehead MR, LanfearR, Mitchell RJ, Karron JD. Plant mating systems often vary widely among populations. Front. Ecol.Evol. 2018; 6: 38. https://doi.org/10.3389/fevo.2018.00038.

54. Sagwe RN, Peters MK, Dubois T, Steffan-Dewenter I, Lattorff HMG. Insect pollination and pollinator supplementation enhances fruit weight, quality, and marketability of avocado (*Persea americana*). Arthropod-Plant Interac. 2023; 17(6): 753-763. https://doi.org/10.1007/s11829-023-09996-3.

55. Wisz MS, Pottier J, Kissling WD, Pellissier L, Lenoir J, Damgaard CF, Dormann CF, Forchhammer MC, Grytnes JA, Guisan A, Heikkinen RK, Hoye TT, Kuhn I, Luoto M, Maiorano L, Nilsson MC, Normand S, Ockinger E, Schmidt NM, Termansen M, Timmermann A, Wardle DA, Aastrup P, Svenning JC The role of biotic interactions in shaping distributions and realised assemblages of species: implications for species distribution modelling. Biol. Rev. 2012; 88(1): 15-30. https://doi.org/10.1111/j.1469-185X.2012.00235.x.

56. Gerard M,Vanderplanck M, Wood T, Michez D. Global warming and plant–pollinator mismatches. Emerg. Top. Life Sci. 2020; 4 (1): 77-86. https://doi.org/10.1042/ETLS20190139.

Legends to Figures

Figure 1. Map of the study area showing study sites along an altitudinal range.

Figure 2. Interaction of insect pollinators of *Berberis lycium* across study sites along altitudinal gradient.

Figure 3. Representing specimens of **v**arious insect pollinators on *Berberis lycium*flowers at different altitudinal range of the study area: A) *Apis* sp., B) *Episyrphusbalteatus,* C) *Apis cerena*, D) *Allograpta* sp., E) *Eristaliscerealis,* F) *Musca domestica,* G) *Dysdercus*sp, H) *Calliphora vomitoria,* I) *Pieris* sp., J) *Bombus trifasciatus,* K) *Calliphora* sp., L)*Rhingia* sp., M) *Chrysotoxumbaphyrum*N) *Eupeodesluniger,* O)*Altica* sp., P)*Coccinellaundecimpunctata*, Q) *Sarcopha*sp., R)*Praezygaenacaschmirensis,* S) *Syritta* sp., T) *Eristalistenax,* U) *Lucilia* sp., V) *Plecia* sp., W) *Formica fusca,* X) *Eristalis* sp., Y) *Eristalinustaeniops*, Z) *Episyrphusviridaureus,* a) *Camponotuspennyslyvanicus,* b) *Aphidoidea,* c)*Aglaiscaschmeriensis,* d) *Coccinellaseptempunctata*, e) *Heliophorusmoorei,* f) *Celastrina*sp, g*) Heliophorus sena*, h*) Heliophorus* sp., i) *Cyrestisthyodamas,* j)*Celastrinaargiolus.*

Figure 4. Activities recorded of different pollinators' orders at different altitudes, A. Foraging behaviour, B. Foraging speed, C. Index of visitation rate, D. Index of visiting efficiency. The best-fitting regression line is represented by the coloured solid line, while the 95% confidence interval of regression line is shown by the shaded areas.

Figure 5. Activities recorded of combined pollinators' orders at different altitudes, A. Foraging behaviour, B. Foraging speed, C. Index of visitation rate, D. Index of visiting efficiency. The best-fitting regression line is represented by the blue solid line, while the 95% confidence interval of regression line is shown by the shaded areas.

Figure 6. Relative abundance of different pollinators' orders at different altitudes. The best-fitting regression line is represented by the coloured solid line, while the 95% confidence interval of regression line is shown by the shaded areas.

Figure 7. Change in reproductive output along altitudinal gradient. a) fruit set (PFS; %), b) seed set (PSS; %), The shape in violin plots at 95% confidence, indicates the data of reproductive output. The violin plots are escorted by whisker boxplots, in which the average reproductive output for each parameter is shown by black lines.

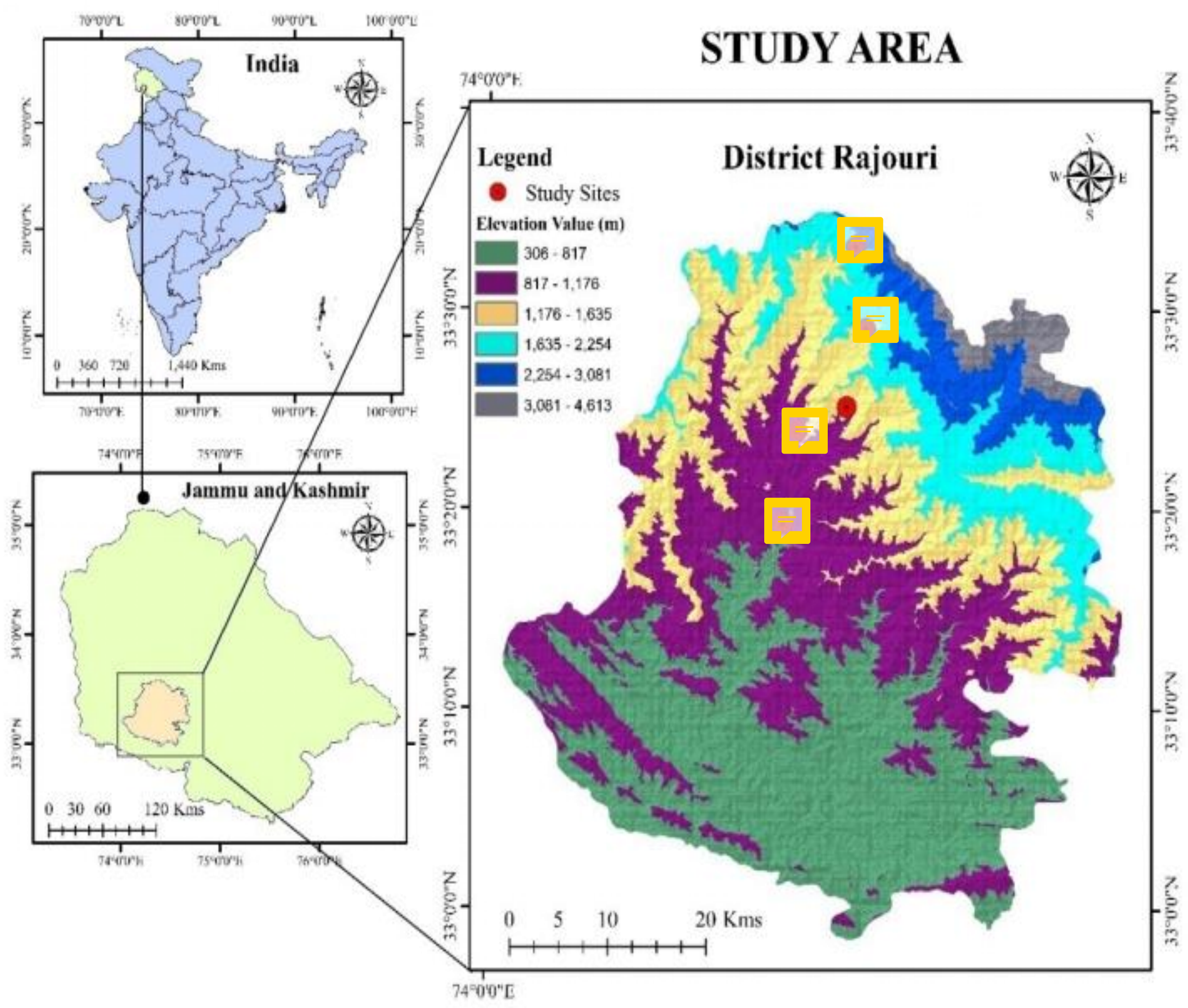

Figure 1. Map of the study area showing study sites along an altitudinal range.

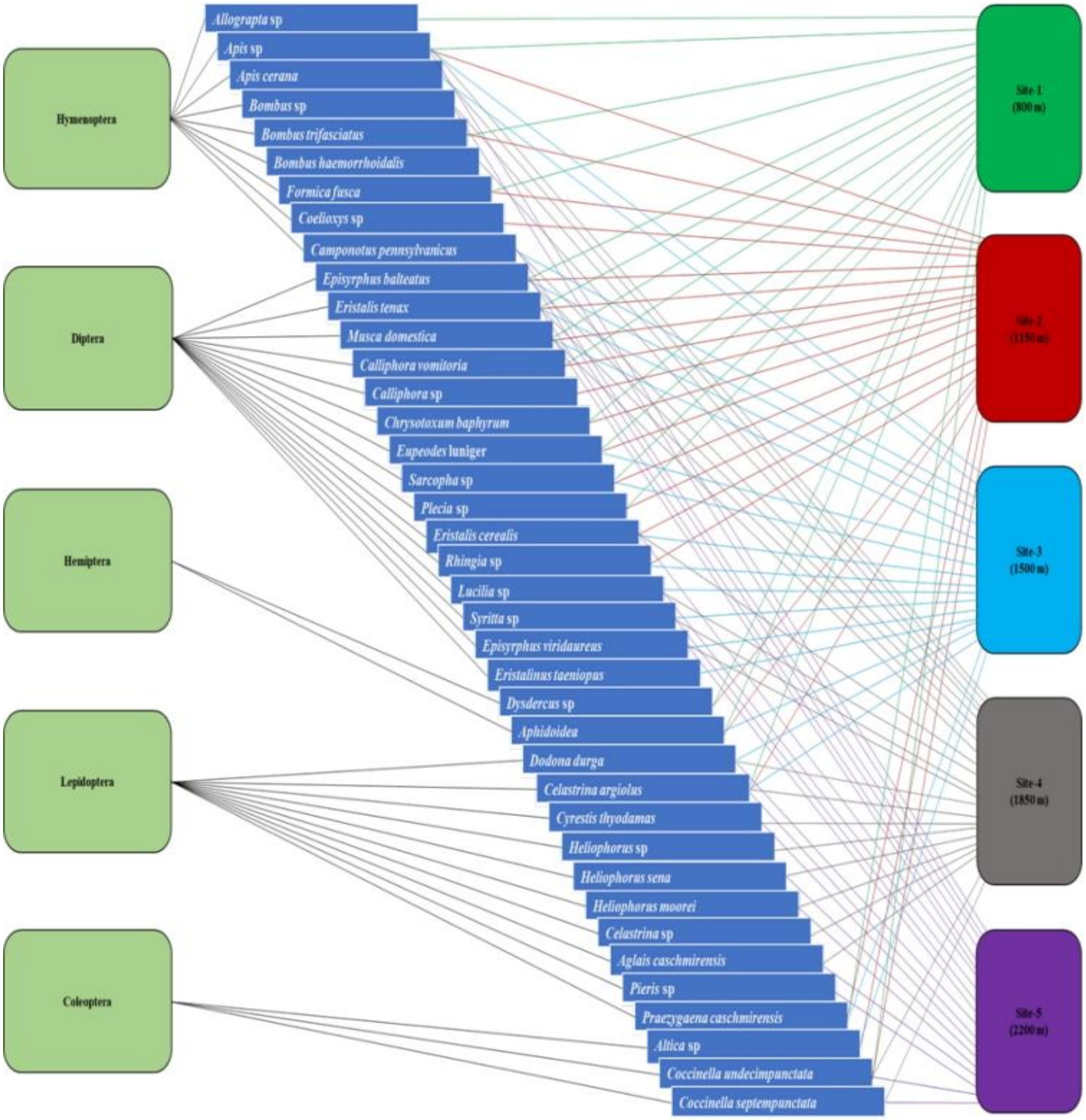

Figure 2. Interaction of insect pollinators of *Berberis lycium* across study sites along altitudinal gradient.

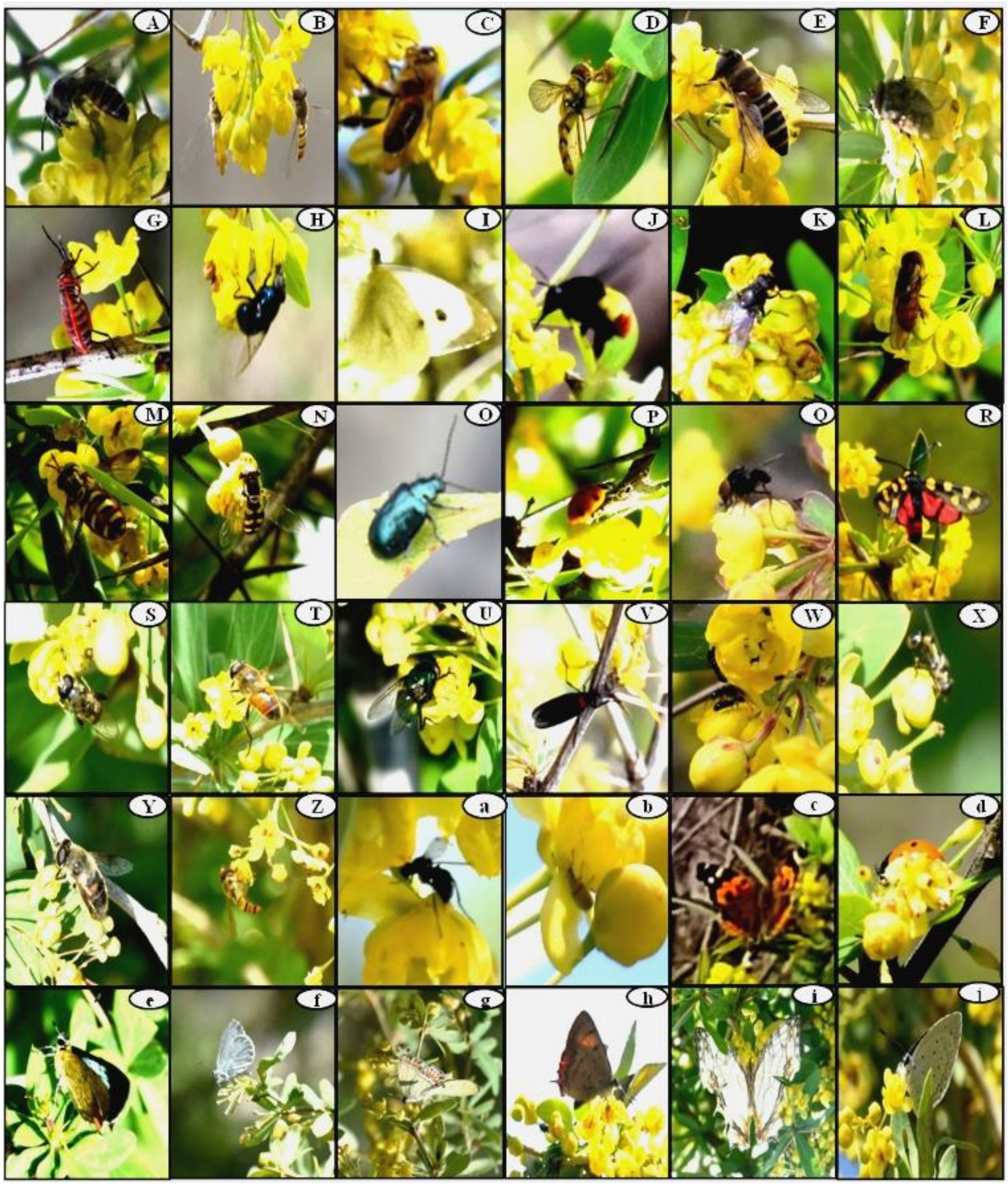

Figure 3. Representing specimens of **v**arious insect pollinators on *Berberis lycium* flowers at different altitudinal range of the study area: A) *Apis* sp., B) *Episyrphus balteatus,* C) *Apis cerena*, D) *Allograpta* sp., E) *Eristalis cerealis,* F) *Musca domestica,* G) *Dysdercus* sp, H) *Calliphora vomitoria*, I) *Pieris* sp., J) *Bombus trifasciatus,* K) *Calliphora* sp., L) *Rhingia* sp., M) *Chrysotoxumbaphyrum* N) *Eupeodes luniger,* O) *Altica* sp., P) *Coccinella undecimpunctata*, Q) *Sarcopha* sp., R) *Praezygaena caschmirensis,* S) *Syritta* sp., T) *Eristalis tenax*, U) *Lucilia* sp., V) *Plecia* sp., W) *Formica fusca*, X) *Eristalis* sp., Y)

Eristalinus taeniops, Z) *Episyrphus viridaureus*, a) *Camponotus pennyslyvanicus*, b) *Aphidoidea*, c) *Aglais caschmeriensis*, d) *Coccinella septempunctata*, e) *Heliophorus moorei*, f) *Celastrina* sp, g) *Heliophorus sena*, h) *Heliophorus* sp., i) *Cyrestis thyodamas*, j) *Celastrina argiolus.*

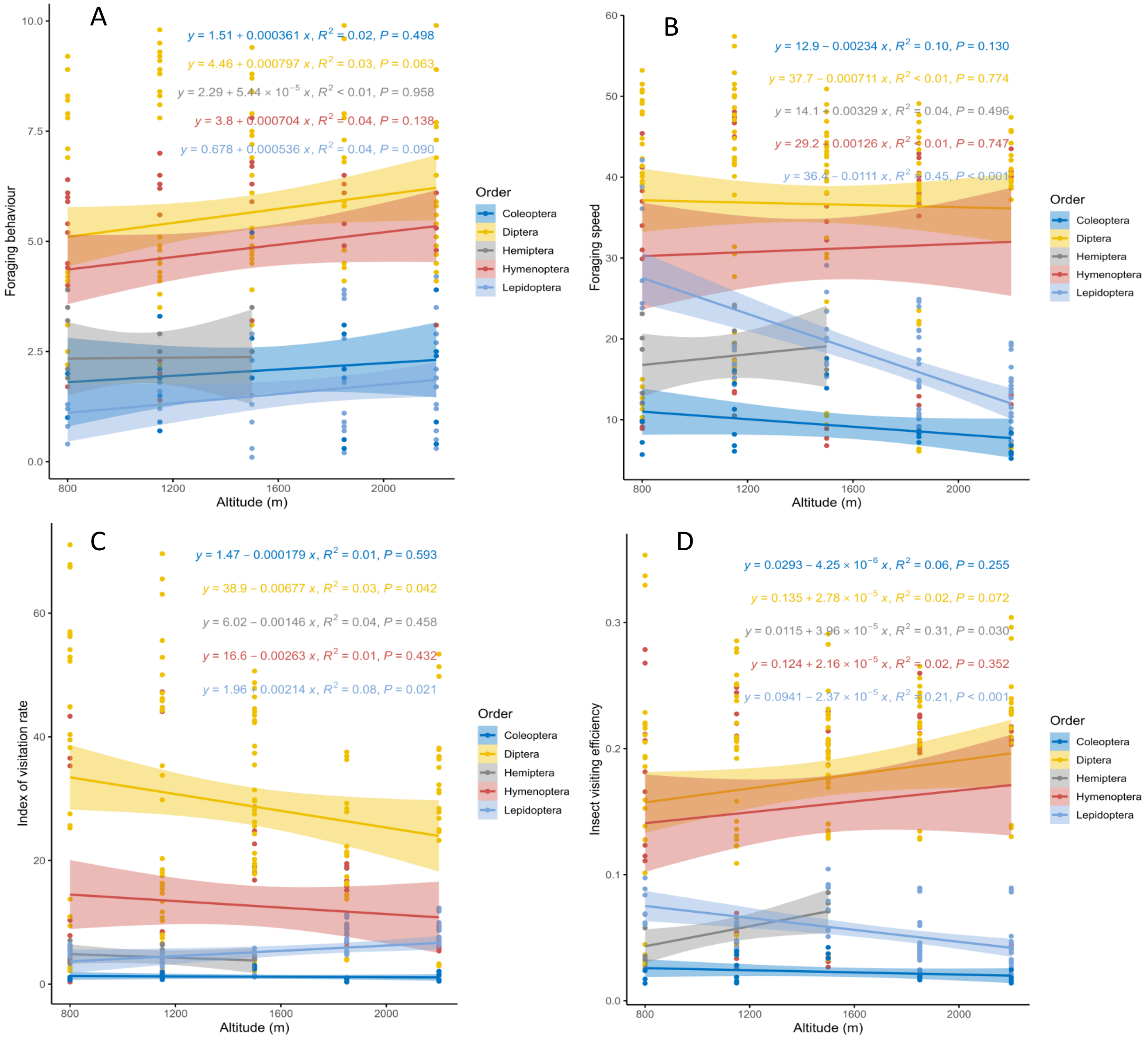

Figure 4. Activities recorded of different pollinators' orders at different altitudes, A. Foraging behaviour, B. Foraging speed, C. Index of visitation rate, D. Index of visiting efficiency. The best-fitting regression line is represented by the coloured solid line, while the 95% confidence interval of regression line is shown by the shaded areas.

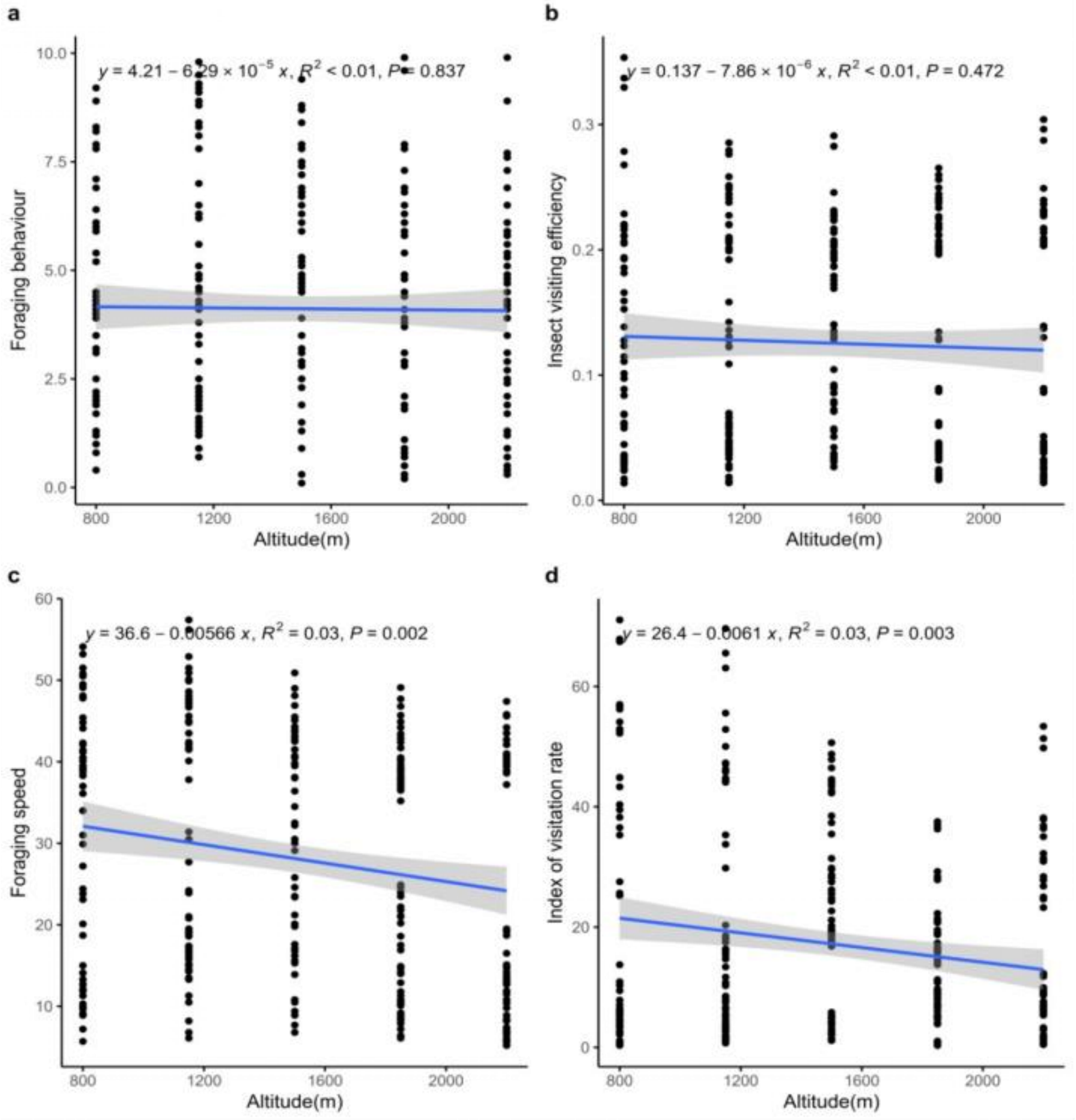

Figure 5. Activities recorded of combined pollinators' orders at different altitudes, A. Foraging behaviour, B. Foraging speed, C. Index of visitation rate, D. Index of visiting efficiency. The best-fitting regression line is represented by the blue solid line, while the 95% confidence interval of regression line is shown by the shaded areas.

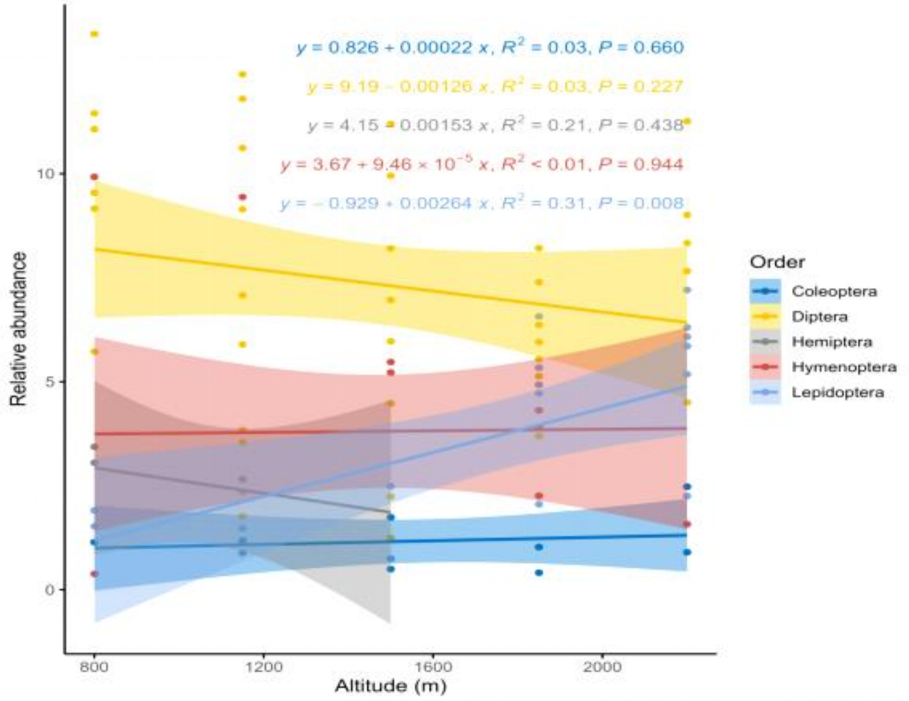

Figure 6. Relative abundance of different pollinators' orders at different altitudes. The best-fitting regression line is represented by the coloured solid line, while the 95% confidence interval of regression line is shown by the shaded areas.

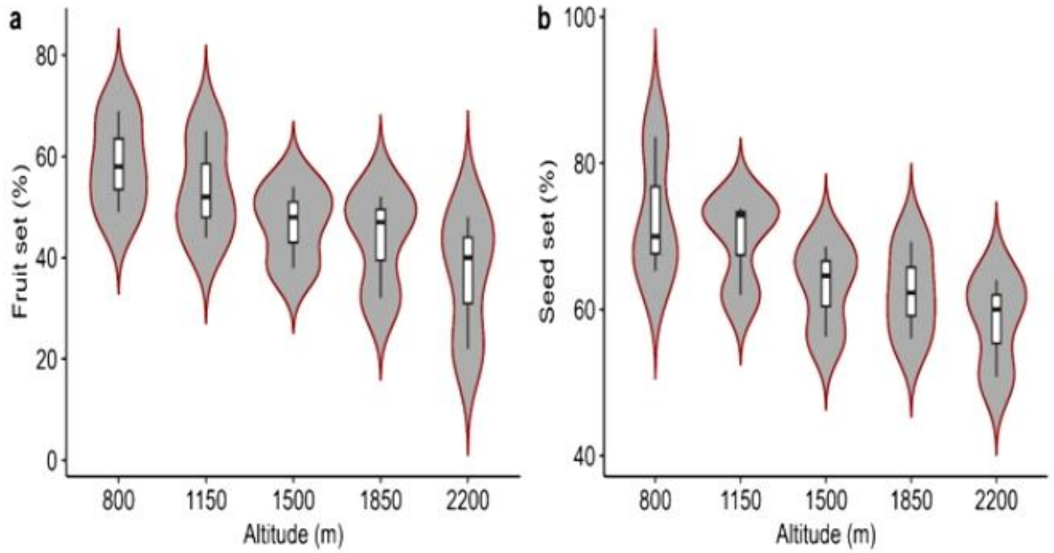

Figure 7. Change in reproductive output along altitudinal gradient. a) fruit set (PFS; %), b) seed set (PSS; %), The shape in violin plots at 95% confidence, indicates the data of reproductive output. The violin plots are escorted by whisker boxplots, in which the average reproductive output for each parameter is shown by black lines.

Supplementary file

Click here to access/download
Supporting Information
Plos_One_Supp Table_1.docx

Highlights

Click here to access/download
Supporting Information
Plos_One_Highlights.docx
