# Supplementary material for: Plant-pollinator interactions along the altitudinal gradient in Berberis lycium royle: An endangered medicinal plant of the Himalayan region
Source: PLoS One. 2025 May 7;20(5):e0310572. doi: 10.1371/journal.pone.0310572 (PMC12058197; doi:10.1371/journal.pone.0310572)
Supplement: Attachment — Submitted filename: PlosOne_Complience to editors comments.docx [file pone.0310572.s005.docx]

**Compliance to editors’ comments**

**Reviewer#1**

**Comment 1:** The terrestrial ecosystem especially Mountains.

**Response:** The sentence has beencorrected as suggested. (Page no. 2, line no.22)

**Comment 2:** and.

**Response:** Corrected. (Page no. 2, line no. 29)

**Comment 3:** Mountains alone are not considered as ecosystem, it’s a broad term, and composed of terrestrial ecosystems among which Mountains are one of the components.

**Response:** Corrected. (Page no. 2, line no. 44)

**Comment 4:** remove.

**Response:** Extra full stop has been removed from sentence. (Page no. 3, line no. 77)

**Comment 5:** done at.

**Response:** Corrected. (Page no. 4, line no. 121)

**Comment 6:** visiting.

**Response:** Corrected. (Page no. 4, line no. 124)

**Comment 7:** stereo zoom binocular microscope.

**Response:** Corrected. (Page no. 4, line no. 125)

**Comment 8:** A total of.

**Response:** Corrected. (Page no. 6, line no. 200)

**Comment 9:** Documented on.

**Response:** Corrected. (Page no. 6, line no. 200)

**Comment 10:** Arrange the species alphabetically or family-wise.

**Response:** Thank you. The names of the species have been arranged alphabetically. (Page no. 8, Table no. 1)

**Comment 11:** Separate genus and species.

**Response:** Genus has been separated from species. (Page no. 9, Table no. 1).

**Reviewer#2**

**Comment 1:** In acknowledgment section it is evident that first author has received funding for this work from CSIR-UGC. Must be mentioned here as well.

**Response:** Thank you for this comment. The funding has been mentioned in the online submission portal.

**Comment 2:** As per ICZN rule it is important to mention the year of the publication at least once in the paper.

**Response:** The year of publication has been mentioned (Zainab et al., 2023). (Page no. 3, line no. 82)

**Comment 3:** Authors need to review the literature of Insect Pollinator interactions and the environmental preference along the attitudinal range as they have missed many important works that have been carried out on insect pollinators along the attitudinal gradient of Himalaya e.g Rather et al. 2023; Singh et al. 2022; Arya and Badoni, 2023; Shrestha et al. 2013 etc.

**Response:** Thank you for valuable comment. The literature review has been revised and the suggested literature have been cited accordingly (Page no. 2)

**Comment 4:** Cite this sentence.

**Response:** The sentence has been cited as suggested. (Page no. 2, line no 47)

**Comment 5:** Geographic pattern.

**Response:** Corrected. (Page no. 2, line no. 49)

**Comment 6:** In my opinion it is not poorly studied. Good amount of work has been carried out. Authors again need to thoroughly review the published work.

**Response:** The sentence has been revised with relevant citations. ((Page no. 3, line no. 69-71)

**Comment 7:** Cite the most recent reference for the global species number of Berberidaceae.

**Response: T**he most recent reference has been cited (Hsieh et al., 2022). (Page no. 3, line no. 80)

**Comment 8:** Cite it.

**Response:** The sentence has been cited. (Page no. 3, line no. 81)

**Comment 9:** Cite.

**Response:** The sentence has been cited. (Page no. 3, line no. 83)

**Comment 10:** Cite.

**Response: We have added** citation. (Page no. 3, line no. 84)

**Comment 11:** Citation is lacking in most of the concrete statements.

**Response: We have revised** and added citations. (Page no. 3, line no. 86, 89, 93)

**Comment 12:** Authors must add note on the pollination mechanism of *B. lycium* whether the plant is self-pollinated or cross-pollinated. This will be much helpful to the readers.

**Response:** Brief note on pollination mechanism of *B. lycium* were already added in the MS as the plant is cross-pollinated (entomophilous). (Page no. 3, line no. 93)

**Comment 13:** revealed.

**Response:** Corrected. (Page no. 3, line no. 97)

**Comment 14:** variation? rewrite this sentence. We revised the sentence.

**Response:** We have revised the sentence. (Page no. 4, line no. 101)

**Comment 15:** From January upto May the Pirpanjal is having extreme cold conditions with snowfall all around. Insect activity all the high-altitude regions generally starts from June-September. Authors must provide the name of the localities, along with the altitude to make this study more authentic and relevant.

**Response:** The authors are thankful for this comment. The name of the localities along with altitude has been added in the text. (Page no. 4, line no. 110)

**Comment 16:** Did the authors submit the collection in this Museum. If yes they must provide the accession number of each sample.

**Response:** The collection is not submitted yet in the Museum. (Page no. 5, line no. 128)

**Comment 17:** The noctural study must be cited? This procedure seems inadequate since the light could have attracted pollinators towards it and thus error in data.

**Response:** The nocturnal study has been cited. We acknowledge your concern about the attraction of nocturnal insects to light. However, we only observed insect pollinators that were already present on B. lycium flowers, if any. Due to the absence of nocturnal insect visitation on the flowers, we confined our study accordingly. (Page no. 4, line no. 132)

**Comment 18:** How modularity changes with different altitudes can provide more meanings to this paper.

**Response:** We agree with your valuable comment that modular changes across different altitudes are important in establishing a meaningful context for the work presented. Therefore, key parameters reflecting this modularity have been highlighted in the first and second paragraphs of the introduction section.

**Comment 19:** Give details about the R package used for the analysis.

**Response:** Thank you for this comment. The details of the packages used has been added in the data analysis section in the highlighted text. (Page no. 6, line no. 175, 177, and 183)

**Comment 20:** I wonder, is this the result of the current study or it is already observed phenological traits of this particular plant species. This part must be omitted from the Results section as its already studied traits of *B. lycium*.

**Response:** The phenological traits of *B. lycium* have already been observed in previous studies. However, in the current study, we not only observed these traits again but also examined how they varied along the altitudinal gradient. Therefore, we have included phenological traits in this study. (Page no. 6, line no. 189)

**Comment 21:** How they determine this number? Generally, we use the mark and recapture method. How authors determined it.

**Response:** We determined this number by randomly selecting 15-30 inflorescences on each of 30 B. lycium plants at each site, with the plants spaced approximately 5 meters apart. The plants were visited for about an hour, with intervals of 5 minutes between each plant, once a week on sunny days. A visual count of individual insects was conducted for 2 minutes on each selected plant, and this was repeated during each sampling hour of the day (Badoni and Arya, 2020). (Page no. 7)

**Comment 22:** Not all samples have been studied upto the species level. Some samples are identified upto genus level only. Add upto the species level identification also.

**Response:** Thank you for this comment. We made every effort to identify all the captured specimens to the species level. However, some insects could only be identified to the genus level. **(**Page no. 6)

**Comment 23:** What is the ecological significance of this study. Since in Fig 2 you have shown the insect network associated with the *B. lycium* along the altitudinal gradient. In order to analyse and interpret this study authors must take the help from already published work on network analysis like Rather et al. (2023); Basu et al. (2022), and similar published work.

**Response:** We greatly appreciate the suggested literature (Rather et al. 2023; Basu et al. 2022). However, in this research, we observed how the single plant species (B. lycium) is pollinated in different months at various altitudes, and which insect species are either common or unique across these altitudes by studying their activities. (Page no. 8, Figure no. 2)

**Comment 24:** They collect pollen and feed on nectar also.

**Response: Thank you, we have** revised the same. (Page no. 8, Table no. 1).

**Comment 25:** See the above comment on *Bombus.*

**Response: Thank you, we have** revised the same. (Page no. 8, Table no. 1).

**Comment 26:** We generally co-relate our work with the previous works on the insect population declines. Did authors find any such previous work on the decline of insect pollinators along the study region.

**Response:** Thank you. We were unable to find any previous work in the studied region that reports a decline in insect populations with increasing latitude (Page no. 17)

**Comment 27:** In online portal data submission, authors have submitted that no funding was available for this study.

**Response:** Thank you for this comment. The funding support has been acknowledged, but it is not currently available. Therefore, we have submitted the work without any available funding. (Page no. 18)

**Comment 28:** Most of the insect pictures are blurred in this paper and it is impossible to identify them upto the species level. Authors must use good quality pictures in this paper as whole of the data analyses are dependent on the proper species identification.

**Response:** Thank you for this comment. The insect pictures have been replaced with higher-quality images. We made every effort to capture clear pictures of insects while they were foraging. However, the captured insects were identified in the laboratory by expert taxonomists. (Page no. 10, Figure No. 3).

**End**
